# Aptamers: precision tools for diagnosing and treating infectious diseases

**DOI:** 10.3389/fcimb.2024.1402932

**Published:** 2024-09-25

**Authors:** Swathi Sujith, Rajalakshmi Naresh, B. U. Srivisanth, Anusree Sajeevan, Shobana Rajaramon, Helma David, Adline Princy Solomon

**Affiliations:** Quorum Sensing Laboratory, Centre for Research in Infectious Diseases (CRID), School of Chemical and Biotechnology, SASTRA Deemed to be University, Thanjavur, India

**Keywords:** aptamers, SELEX, biosensor, therapeutics, diagnosis, bacteria, virus, infectious disease

## Abstract

Infectious diseases represent a significant global health challenge, with bacteria, fungi, viruses, and parasitic protozoa being significant causative agents. The shared symptoms among diseases and the emergence of new pathogen variations make diagnosis and treatment complex. Conventional diagnostic methods are laborious and intricate, underscoring the need for rapid, accurate techniques. Aptamer-based technologies offer a promising solution, as they are cost-effective, sensitive, specific, and convenient for molecular disease diagnosis. Aptamers, which are single-stranded RNA or DNA sequences, serve as nucleotide equivalents of monoclonal antibodies, displaying high specificity and affinity for target molecules. They are structurally robust, allowing for long-term storage without substantial activity loss. Aptamers find applications in diverse fields such as drug screening, material science, and environmental monitoring. In biomedicine, they are extensively studied for biomarker detection, diagnostics, imaging, and targeted therapy. This comprehensive review focuses on the utility of aptamers in managing infectious diseases, particularly in the realms of diagnostics and therapeutics.

## 1 Introduction

Pathogens, such as bacteria, fungi, viruses, or parasitic protozoa transmitted throughout populations, are typically the source of infectious diseases, some recognized as potentially fatal (Wan et al., 2021; Krüger et al., 2021; Zhang et al., 2021). Infectious diseases continue to be a significant global public health concern, representing the primary causes of morbidity and mortality (Cohen, 2000). Similar signs and symptoms are common among numerous diseases, and the diagnosis, treatment, and management of infectious diseases may face significant difficulties due to the emergence of novel pathogens as well as the reappearance and rise of previously identified pathogen variations (Chen et al., 2022b) (Wan et al., 2021). The rise of antimicrobial resistance can be attributed to the improper or empirical use of antibiotics in the treatment of infections. This underscores the need for careful and evidence-based antibiotic management in addressing infectious diseases (Fair and Tor, 2014; Rahbi et al., 2023). While laboratory testing, imaging scans, and biopsies based on clinical signs and epidemiological data have been successfully used to identify infections, these conventional procedures are either labor-intensive or highly complex (Wan et al., 2021; Zhang et al., 2021).

Therefore, it is imperative to develop new, quick, and precise diagnostic and therapeutic techniques to address the issues of drug resistance and anti-microbial resistance (Krüger et al., 2021). Aptamer-based diagnostic technologies are among the diagnostic approaches that are rapidly being employed for molecular disease diagnosis due to their cost-effectiveness, sensitivity, specificity, and convenience (Wan et al., 2021). The Latin word “aptus”, which means “to fit,” and the Greek word “meros”, which means “region,” are the sources of the word “aptamer” (Ku et al., 2015). Aptamers, single-stranded RNA or DNA oligonucleotide sequences with a length of approximately 25–80 bases, are the nucleotide counterparts of monoclonal antibodies. They may bind target molecules with high affinity and specificity, demonstrating the nucleic acid’s multifunctional nature (Ni et al., 2021). Aptamers offer a range of benefits, such as being cost-effective, exhibiting minimal batch-to-batch variation, demonstrating low immunogenicity, and possessing a small size for improved tissue penetration (Otte et al., 2022). Despite their potential, aptamers are constrained by their rapid clearance through renal filtration and susceptibility to nuclease hydrolysis, leading to a very short half-life *in vivo* (Kovacevic et al., 2018; Ni et al., 2021). In response to these limitations, several techniques have been developed to extend the half-life. These include PEGylation for sustained action, modification of sugar ring or base, phosphodiester linkage, and 3′ end capping with inverted thymidine (Ni et al., 2017). Due to their structural stability, aptamers can be manufactured in large quantities and stored for extended periods without significant activity loss (Srivastava et al., 2021).

Various aptamers have been developed against various targets such as hormones, viruses, metal ions, proteins, viruses, and bacteria (Zhou and Rossi, 2017; Shraim et al., 2022). These complexes form stable and specific targets with dissociation constants in the nanomolar range. Additionally, aptamers have a greater target range, it is easier to regenerate, substantially smaller, and is neither poisonous nor immunogenic (García-Recio et al., 2016; Zheng et al., 2015; Roxo et al., 2019). New aptamer reports are released nearly daily due to their broad applicability. A specific database has been built (https://sites.utexas.edu/aptamerdatabase) to classify the aptamer-related data and enable access to information about various existent aptamers (Askari et al., 2024). Aptamers have drawn a lot of interest in the biomedical community due to their unique qualities and wide applications in a variety of sectors, including drug screening, material science, and environmental monitoring (Chen et al., 2022a).

Aptamers have been extensively studied and developed over the past 20 years by researchers in several biomedical fields, including biomarker detection, diagnostics, imaging, and targeted therapy. Aptamers that are now utilized in cancer treatment can bind to and block the immunoregulatory components of carcinogenesis, which are particular to molecular targets that are characteristic of various diseases. In December 2004, the US Food and Drug Administration approved pegaptanib (Macugen), the first medication based on aptamer technology, for the treatment of age-related macular degeneration (Adachi and Nakamura, 2019). Despite the lack of new aptamers approved for clinical use, there is promising progress in the development of aptamers for blood disorders, with several of them currently undergoing different stages of clinical trials and proof-of-concept investigations (Aljohani et al., 2022). Aptamers demonstrate a wide range of applications, highlighting their versatile nature in the field of infectious diseases. Thus, the review provides an in-depth insight into the general mechanism of aptamer selection and its applications in the diagnostic and therapeutic fields. Furthermore, it addresses recent advances and challenges in the field of aptamers, aiming to inspire further exploration of aptamer-based approaches in combating infectious diseases.

## 2 Mechanisms of aptamer selection

The process of aptamer selection includes a range of methodologies designed to identify nucleic acid sequences that can bind specific target molecules with high affinity and specificity (Kinghorn et al., 2017). Both SELEX (Systematic Evolution of Ligands by Exponential Enrichment) and non-SELEX approaches are used to refine methods. SELEX employs iterative rounds of selection, in which a nucleic acid library interacts with the target molecule under controlled conditions, to enhance sequences with optimal binding properties (Uemachi et al., 2021). Contrastingly, Non-SELEX methods steer clear of traditional scaffold-based approaches, opting instead for innovative strategies to bolster aptamer stability, specificity, and interaction dynamics (Kong and Byun, 2013). These diverse methodologies empower researchers to confidently tailor aptamer selection processes according to the specific requirements of their applications, from diagnostics to therapeutic interventions.

### 2.1 Systematic evolution of ligands by exponential enrichment

SELEX is a method used to derive aptamers from a pool of nucleotide sequences that exhibit high affinity and selectivity (Chen et al., 2016). The process involves several key steps to select aptamers through a repetitive cycle of amplification and enrichment. Initially, a large and diverse library of nucleic acid sequences (DNA or RNA) is synthesized and incubated with the target molecule in an appropriate buffer at a specific temperature (Sun et al., 2014). The partitioning or the eluting steps involves removal of the unbound nucleotide by chromatography, electrophoresis or filtration (Dong et al., 2018). A low ratio of nucleic acid sequences to the target molecule is used, ensuring effective binding. The aptamer-target complexes are then separated from unbound sequences using techniques such as capillary electrophoresis (Hamedani and Müller, 2016), magnetic bead separation (Yüce et al., 2015), and flow cell methodologies (Gopinath, 2007).

**Figure 1:**
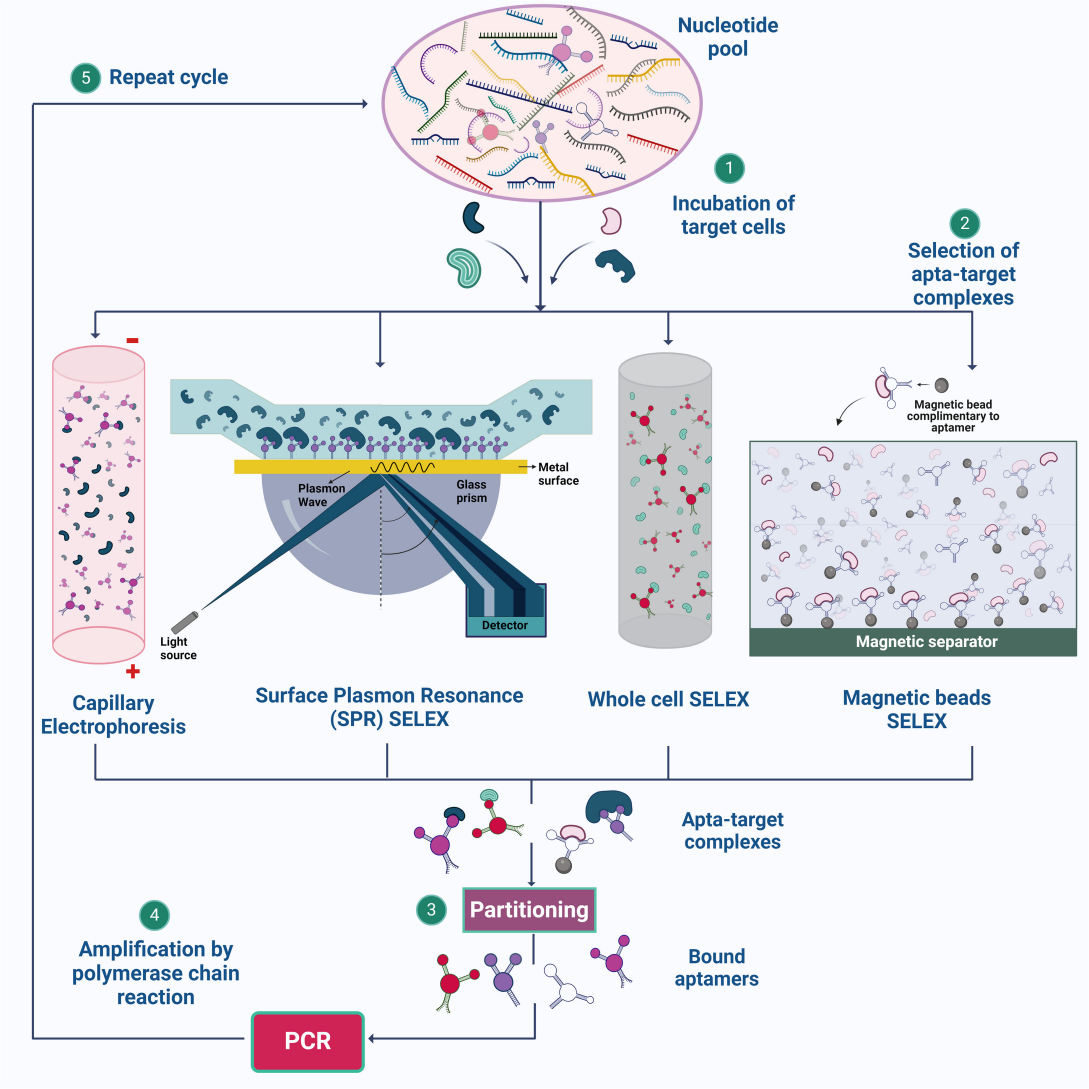
Illustration of SELEX strategies for aptamer synthesis. (1) The process begins with the preparation of a nucleotide pool, which is then incubated with target cells. (2) Various SELEX methods, such as Capillary Electrophoresis SELEX, SPR-SELEX, Whole Cell SELEX, and Magnetic Beads SELEX, are employed to facilitate the selection of aptamer-target complexes. (3) & (4) These complexes are isolated, and the bound aptamers are subsequently amplified by PCR. (5) The cycle is repeated multiple times to enhance the specificity and affinity of the aptamers for their targets. Created using Biorender.com.

The bound sequences are eluted from the target and amplified using PCR for DNA aptamers or reverse transcription followed by PCR for RNA aptamers, creating a new, enriched library. These processes are repeated for several rounds, typically 8-15, to enhance the prevalence of high-affinity species, which eventually dominate the library (Zhou and Rossi, 2017) (**Figure 1**). After multiple rounds of selection, the enriched library is cloned and sequenced to identify individual aptamer sequences, which are then validated for their binding performance. Through these iterative rounds, SELEX effectively isolates aptamers that can bind to specific target molecules with high affinity and specificity. However, a common drawback of aptamers derived from traditional SELEX methods is poor or nonspecific detection performance in diagnostic applications (Bakhtiari et al., 2021). To overcome these shortfalls, different methodologies are incorporated over conventional SELEX, some of which are discussed below.

#### 2.1.1 Magnetic beads SELEX

Magnetic SELEX is a method that is commonly employed as this method offers ease in the separation of the target and nucleotide sequence easily from the remaining reaction mixture by employing a magnet (Yüce et al., 2015). When the DNA sequence binds with the target molecule, the mixture is now added with magnetic beads coated with a molecule that can selectively bind with the nucleic acid sequence attached to the target molecule. The elution of the bound nucleic acid sequences from the magnetic beads is achieved by altering the buffer’s properties, applying heat, or utilizing other methods that hinder the nucleic acids’ binding to the magnetic beads (Komarova and Kuznetsov, 2019). In previous research, the isolation of Metamitron (MTM) aptamers using magnetic-bead SELEX has been successful. MTM, a widely used herbicide in agriculture, has been the subject of a thorough investigation. It is important to note that even with significant exposure, the negative health effects on humans are minimal. Following ten rounds of screening, high-throughput sequencing successfully identified six outstanding candidate aptamers with remarkable affinity and specificity (Xie et al., 2022).

#### 2.1.2 Capillary electrophoresis

Apart from using traditional gel electrophoresis, capillary electrophoresis (CE) is employed to derive aptamer candidates on the metrics of sizes and charge; under the electric field, capillary electrophoresis can separate molecules as tiny as porphyrin30 (Yüce et al., 2015). When performing CE-SELEX, the target molecules are subjected to incubation with the random library in free solution, and the resulting combination of free target molecules, target-ssDNA complexes, and free ssDNA is then fed into a capillary column, then split apart using a high voltage. Taking a sample of the output fraction at the designated retention time, target-bond ssDNA provides the chance to collect DNA aptamers that bind to a specific target (Hamedani and Müller, 2016). Demonstrating the perspective of CE-SELEX for small-molecule targets in just four rounds. Small-molecule targets are anticipated to alter the mobility of the complex only slightly from the nonbinding sequences, leading to only partial separation of the bound and unbound sequences. However, even if just a tiny amount of the complex can be recovered, adequate enrichment can be accomplished since nucleic acids can be exponentially amplified by polymerase chain reaction (PCR). Additionally, repeated recurrent rounds of enrichment can eventually lead to the evolution of an abundant pool with high quality, even in the situation of separation with poor resolution (Yang and Bowser, 2013).

When CE-SELEX and high throughput sequencing (HTS) gave higher efficiency with faster separation of target-ssDNA complex and free ssDNA in free solution, aptamers can be chosen with relatively fewer rounds of selection thanks to HTS, which offers insight into the sequence evolution during the CE-SELEX process and makes it possible to characterize the entire evolutionary path. This reduces the need for the pool to occupy a consensus sequence and increases selection efficiency (Zhu et al., 2021).

#### 2.1.3 Whole cell SELEX

While the major targets for the other SELEX techniques are highly purified targets, whole cell-SELEX uses a complete cell as the target. The cell-SELEX procedure may aim for extracellular cell surface proteins or unidentified cell structures. This SELEX approach makes it possible to create whole-cell targeting aptamers without much prior information on the cell’s surface proteins, which facilitates the identification of new biomarkers primarily for diagnosis and imaging (Yüce et al., 2015). The whole-cell SELEX method is used to create highly selective aptamers by different rounds of SELEX and counter SELEX. Aptamer can be separated using methods such as flow cytometry, Magnetic-Activated Cell Sorting, Differential Centrifugation, and Label-free methods. Whole-cell SELEX yields aptamers with high affinity and specificity when targeting bacterial surface compounds and live bacterial cells. Flow cytometry is a vital method for identifying target aptamers that bind selectively to cells. The technique overcomes the limitations of whole-cell SELEX by sorting, counting, and detecting fluorescence (Moon et al., 2013). Within flow cytometry techniques, fluorescence-Activated Cell Sorting (FACS) technique offers the ability to simultaneously differentiate and separate cell subpopulations, facilitating the identification of bound and unbound aptamers with specificity along with isolation of functional nucleic acids. By utilizing a sorting device that efficiently separates specific cells based on their fluorescence, FACS streamlines the process of finding aptamers that target different cell types contributing to a better yield of the aptamer candidates. The effectiveness of FACS in SELEX for functional aptamer selection is apparent in its successful separation of *E. coli* cells that produce RNA mimics (Nishimoto et al., 2007; Mayer et al., 2010; Zou et al., 2015). FACS is an effective method for large-scale aptamer screening because it is a fast and accurate technique that can process thousands of cells per second. It can sort cells based on multiple parameters and select aptamers based on their binding to live cells or complex mixtures, which may be more representative of physiological conditions than selections made *in vitro*. The possibility of obtaining high-quality aptamers is increased by the capacity to sort and enrich high-affinity binders from a huge library. FACS employs both positive and negative selection strategy, thereby reducing the experimental steps and experimental errors in the cell SELEX process, hence saves times. DNA Aptamers against Burkitt’s lymphoma cells which exhibit a characteristic phenotype was chosen using positive selection methods (Ohuchi, 2012; Raddatz et al., 2008; Sola et al., 2020). Despite the benefits of the cell-SELEX system, the low aptamer enrichment performance of this technique is caused by the co-expression of several off-target surface indicators and compounds on the target cells (Sun et al., 2014).

#### 2.1.4 Surface plasmon resonance or flow cell SELEX

SPR- SELEX utilizes SPR for the selection process, differentiating it from the other methods. A Randomized library is passed over a surface coated (gold surface) with the target molecule (Yüce et al., 2015). In the library, a diverse range of oligonucleotides interact with the target in various ways. Oligonucleotides demonstrating strong binding will firmly adhere to the target-coated surface, while those with weak binding or unbound sequences will be effectively washed away (Jia et al., 2018). In SPR the nucleic acid sequence bound to the target molecule will be monitored in real time by observing the change in the refractive index on the surface leading to the change in surface plasmon signal (Ferhan et al., 2016). With the help of the above-mentioned steps, specific aptamer candidates are carefully selected and amplified using PCR (Jia et al., 2018).

### 2.2 Non-SELEX methods

SELEX uses a nucleic acid scaffold to develop the aptamer; however, other techniques do not require scaffolds (Reverdatto et al., 2015). For instance, aptamers are produced in the RNase III-deficient *E. coli* HT115(DE3), and 5′- and 3′ ends of the RNA transcript are protected from the RNase using double stranded spacers. This method only required fewer nucleotides than scaffold-based methods like the other different types of SELEX used to avoid RNase activity on the formed aptamer (Zou et al., 2023). PhotoSELEX, featuring photoreactive nucleic acids, confidently enhances control over the selection process. Upon exposure to light, the photoreactive groups confidently form covalent bonds between the selected aptamers and the target molecule, confidently providing a reliable method for identifying and capturing aptamer-target complexes (Brody et al., 1999). Graphene oxide (GO) is composed of carbon atoms arranged in a hexagonal lattice. Its unique properties allow for the immobilization of arbitrary DNA or RNA sequences on its surface, forming an oligonucleotide library with diverse sequences. During the GO-SELEX process, the target molecule interacts with the library-immobilized sequences. In the presence of the target molecule, the immobilized sequences on the GO surface are released and precisely interact with the target. This stage allows for the selection of aptamers with a high affinity for the target molecule (Nguyen et al., 2014; Ding and Liu, 2023). In the Capture-SELEX process, a DNA library is immobilized onto a substrate. The target of interest is then passed through to extract eluted aptamers. Aptamers are specifically chosen using this strategy for solute targets (Boussebayle et al., 2019). These non-SELEX methods provide versatile alternatives, overcoming challenges such as RNase degradation, and enhancing binding affinity through innovative selection techniques.

The aptamers that are selected can be used in various applications. One groundbreaking application is the use of apta-sensors for detecting infectious diseases. These biosensors use aptamers as recognition elements, and they provide fast, sensitive, and specific detection of pathogens. By incorporating aptamers selected through SELEX or Non-SELEX methods, apta-sensors can accurately detect infectious agents, greatly improving diagnostic capabilities. Their versatility and ability to detect a wide range of pathogens make aptasensors extremely valuable tools in epidemiology, healthcare settings, and biodefense (Brosseau et al., 2023).

## 3 Aptamers in diagnostics of infectious diseases

**Figure 2:**
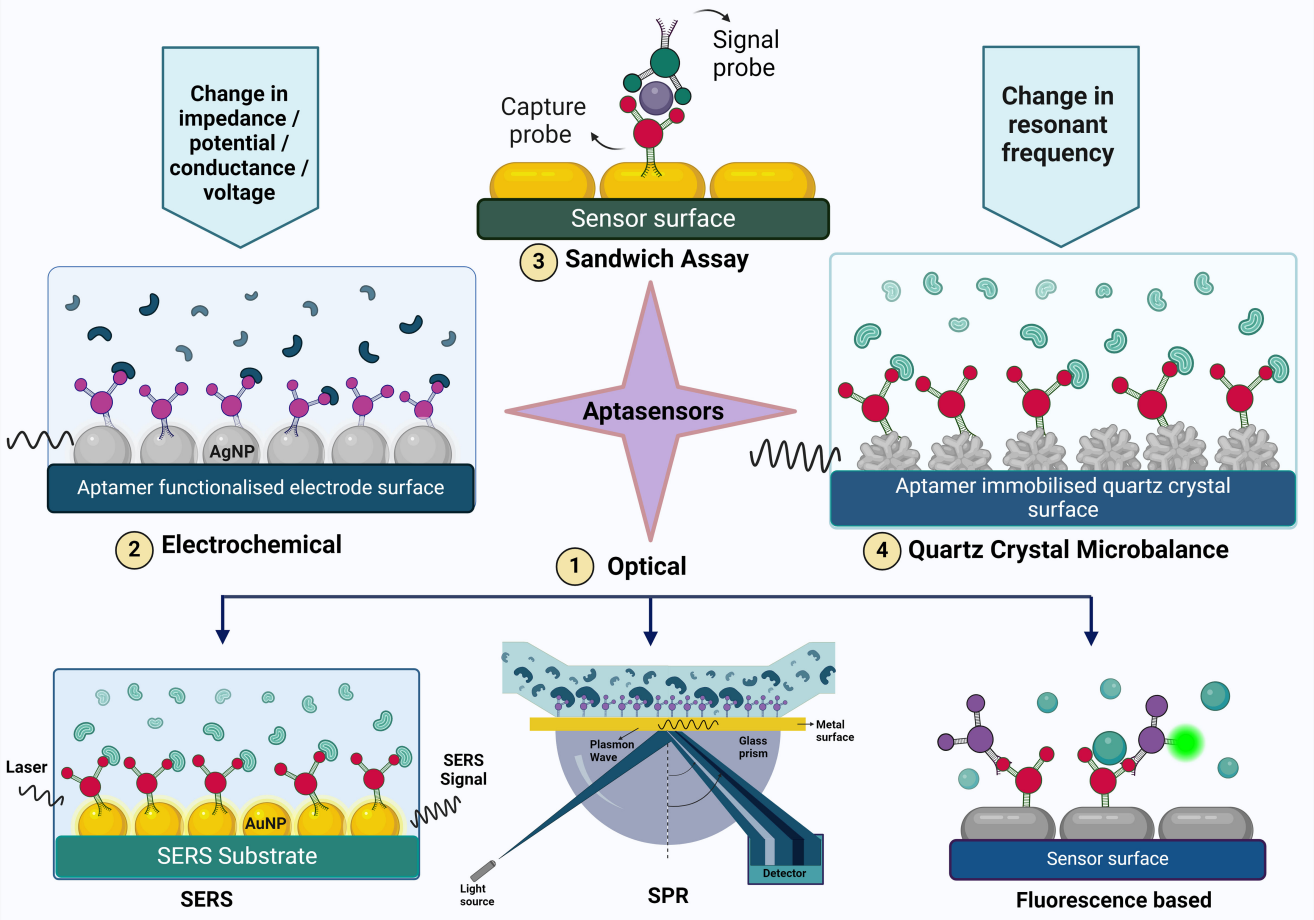
The figure illustrates various aptasensing mechanisms used for detecting target molecules. These mechanisms include (1) Optical sensors (such as Surface-Enhanced Raman Scattering (SERS), Surface Plasmon Resonance (SPR), and fluorescence-based methods), (2) Electrochemical sensors, (3) Sandwich assays, and (4) Quartz Crystal Microbalance (QCM). Each mechanism provides a unique approach to aptamer-based detection, highlighting the versatility and specificity of aptamers in biosensing applications.

Traditional methodologies for detection encompass culture-based techniques and color culture medium approaches. However, these methodologies are encumbered by limitations, necessitating professional expertise, and demanding cumbersome labor and time commitments. The procedural intricacies include pre-enrichment, selective enrichment, and biochemical identification, typically leading to a confirmed outcome after 2-3 days (Bell et al., 2016). Due to the limitations present in these methods, there is a need for more efficient, rapid and accurate diagnostic methods. Immunological assays, such as ELISA and immunosensors, are commonly used for bacterial detection. However, their sensitivity is limited because proteins like immunoglobulins cannot be amplified. Furthermore, nucleic acid-based assays are unable to distinguish between viable and non-viable cells, as DNA can persist in the environment long after cell viability has been lost. This creates a need for a more specific, sensitive, and convenient diagnostic method that can bridge the gap between the detection. Aptamer-based assays are utilized for the detection of pathogens and biomarkers. Aptamers synthesis is rapid compared to the antibody production, and these rapid turnaround time helps in timely diagnosis. Furthermore, they have increased stability and shelf life compared to antibodies and reduced risk of immunogenicity due to ease of modifications that increase the stability, binding affinity and functionality (Ali et al., 2019). These assays enhance detection methods by providing improved specificity and sensitivity even at lower concentrations compared to traditional methods (Aslan et al., 2023). By delivering rapid results, which are ideal for point-of-care settings, this approach enhances diagnostic efficiency across various healthcare applications (Majdinasab et al., 2022). Further, the review delineates a comprehensive analysis of the diverse categories of apta-sensors. (**Figure 2**, **Table 1**).

**Table 1:** Pathogen detection table: Pathogens, aptamer sequences, and detection mechanisms.

S.No.	Aptamer sequence (5’ to 3’)	Type	Organism	Target	Concentration range	LOD (limit of detection)	Methodology	Reference
Bacteria								
1.	S-S- ATCCGTCACACCTGCTCTGTCTGCGAGCGGGGC GCGGGCC CGGCGGGGGATGCGTGGTGTTGGCTCCCGTAT	DNA	*E. coli O157:H7*	Outer membrane proteins	10^1^ to 10^5^ CFU/mL	2.9 × 10^2^ CFU/mL	Impedimetric aptasensor	(Brosel-Oliu et al., 2018)
2.	ATCCGTCACACCTGCTCTGTCTGCGAGCGGGGCGCGGGCCCGGC GGGGGATGCGTGGTGTTGGCTCCCGTAT	DNA	*E. coli O157:H7*	–	500 to 5x10^7^ CFU/mL	250 and 400 CFU/mL, for buffer and milk samples respectively	Eye-based microfluidic aptasensor (EA-Sensor)	(Li et al., 2020)
3.	CCATGAGTGTTGTGAAATGTTGGGACACTAGGTGGCATAGAGC CG-C_6_-SH	DNA	*E. coli*	–	3.2 × 10^1^ to 3.2 × 10^7^ CFU/mL	3.46 CFU/mL	SERS aptasensor	(Ye et al., 2022)
4.	Apt1 (signal probe) A20-CCGGACGCTTATGCCTTGCCATCTACAGAGCAGGTGTGACGG Apt2 (capture probe) biotin-CCGGACGCTTATGCCTTGCCATCTACAGAGCAGGTGTGACGG-3	DNA	*E. coli O157:H7*	–	10 to 10000 CFU/mL	3 CFU/mL	Gold nanobones enhanced ultrasensitive SERS aptasensor	(Zhou et al., 2020)
5.	CAG TCC AGG ACA GAT TCG CGA G-N_45_-CAC GTG GAT TTC ATT CAG CGA TT	ssDNA	*E. coli O157:H7*	–	–	1.46 × 10^3^ CFU/mL	Aptamer-antibody sandwich assay	(Yu et al., 2018)
6.	ATCCAGAGTGACGCAGCA-(N45)- TGGACACGGTGGCTTAGT	DNA	*E. coli O78:K80:H11*	–	10^1^ to 10^6^ CFU/mL	10 CFU/mL	Bridged rebar graphene functionalized impedimetric aptasensor	(Kaur et al., 2017)
7.	ATCCAGAGTGACGCAGCA-(N45)-TGGACACG GTGGCTTAGT	ssDNA	*E. coli O157:H7*	–	10^0^ to 10^5^ CFU/mL	10 CFU/mL	Electrochemical aptasensor using boron-carbon nanorods decorated by nickel nanoparticles	(Kaur et al., 2020)
8.	P-CCG GAC GCT TAT GCC TTG CCA TCT ACA GAG CAG GTG TGA CGG	DNA	*E. coli O157:H7*	LPS of *E. coli* O157:H7	–	3 CFU/mL	Zirconium-based metal−organic frameworkTi_3_C_2_T_x_ nanosheet based faraday cage-type electrochemical aptasensor	(Dai et al., 2022)
9.	SH-ATC CGT CAC ACC TGC TCT GTC TGC GAG CGG GGC GCG GGC CCG GCG GGG GAT GCG TGG TGT TGG CTC CCG TAT	DNA	*E. coli O157:H7*	–	500 to 5000 CFU/mL	116 CFU/mL	MoS_2_ nanosheets-based label-free colorimetric aptasensor	(Li et al., 2023)
10.	TATGGCGGCGTCACCCGACGGGGACTTGACA TTATGACAG	DNA	*Salmonella enterica*	–	10^8^ to 10^1^ CFU/mL	10^1^ CFU/mL	Reduced graphene oxide-titanium dioxide nanocomposite-based electrochemical aptasensor	(Muniandy et al., 2019)
11.	–	dsDNA	*Salmonella typhimurium*	–	10 to 10^5^ CFU/mL	6 CFU/mL	Aptasensor based immuno-HCR-SERS method with dual signal amplification capability	(Li et al., 2021)
12.	C_6_-NH_2_-CTGTCATAAT GTCAAGTC	CdTe QD-labeled ssDNA2	*Salmonella* *typhimurium*	Outer membrane proteins	10 to 10^10^ CFU/mL	1 CFU/mL	Aptamer-based fluorescence assay	(Ren et al., 2019)
13.	ATTAGTCAAGAGGTAGACGCACATAAGGGGTCTGGTGTCGGGCCGC GGGTCAGGGGGGTAAGGGATTCTGGTCGTCGTGACTCCTAT	ssDNA	*Salmonella paratyphi A*	–	–	10 CFU/mL	FRET based aptasensor	(RM et al., 2020)
14.	Apt1 botin- GAGGAAAGTCTATAGCAGAGGAGATGTGTGAACCGAGTAA Apt2 CTCCTCTGACTGTAACCACGGAGTTAATCAATACAAGGCGGGAACA TCCTTGGCGGTGCCGCATAGGTAGTCCAGAAGCC	ssDNA	*Salmonella typhimurium*	–	3.3 × 10^1^ to 3.3 × 10^6^ CFU/mL	33 CFU/mL in pure culture and 95 CFU/mL in spiked milk	Colorimetric sensor based on dual aptamers - the absorbance intensity ratio (A_523_/A_650_) for quantitative analysis of various concentrations of bacteria	(Chen et al., 2021a)
15.	TAT GGC GGC GTC ACC CGA CGG GGA CTT GAC ATT ATG ACA G	DNA	*Salmonella* *typhimurium*	–	10 to 10^5^ CFU/mL	4 CFU/mL	SERS using spiny gold nanoparticles (SGNPs)	(Ma et al., 2018)
16.	Apt 1 SH-AGTAATGCCCGGTAGTTATTCAAAGATGAGTAGGAAAAGA Apt2 ROX-AGTAATGCCCGGTAGTTATTCAAAGATGAGTAGGAAAAGA	DNA	*Salmonella* *typhimurium*		15 to 1.5 × 10^6^ CFU/mL	15 CFU/mL	SERS-*S.typhimurium* specifically interacted with the aptamers to form Au@Ag-apt 1-target-apt 2-ROX sandwich-like complexes.	(Duan et al., 2016)
17.	HS-TATGGCGGCGTCACCCGACGGGGACTTGACATTATGACAG	ssDNA	*Salmonella enterica*		10 to 10^5^ CFU/mL	1.223 CFU/mL	Competitive voltammetric aptasensor based on electrospun carbon nanofibers-gold nanoparticles modified graphite electrode	(Fathi et al., 2020)
18.	NH_2_-TTT GGT CCT TGT CTT ATG TCC AGA ATG CGA GGA AAG TCT ATA GCA GAG GAG ATG TGT GAA CCG AGT AAA TTT CTC CTA CTG GGA TAG GTG GAT TAT	DNA	*Salmonella* *typhimurium*		10^1^ to 10^8^ CFU/mL	6 CFU/mL	Diazonium-based impedimetric aptasensor	(Bagheryan et al., 2016)
19.	NH_2_-TAT GGC GGC GTC ACC CGA CGG GGA CTT GAC ATT ATG ACA-G	DNA	*Salmonella*		75 to 7.5×10^5^ CFU/mL	25 CFU/mL	Impedimetric aptasensor using a glassy carbon electrode modified with an electrodeposited composite consisting of reduced graphene oxide and carbon nanotubes	(Jia et al., 2016)
20.	SH-GCA ATG GTA CGG TAC TTC CTC GGC ACG TTC TCA GTA GCG CTC GCT GGT CAT CCC ACA GCT ACG TCA AAA GTG CAC GCT ACT TTG CTA A	DNA	*S. aureus*		10^1^ –10^7^ CFU/mL	1 CFU/mL	Dual recognition aptasensor	(El-Wekil et al., 2022)
21.	SH-TCG GCA CGT TCT CAG TAG CGC TCG CTG GTC ATC CCA CAG CTA CGT C	DNA	*S. aureus*		1.2×10^1^ to 1.2×10^8^ CFU/mL	1 CFU/mL	Electrochemical aptasensor using Au nanoparticles/carbon, nanoparticles/cellulose, nanofibers nanocomposite	(Ranjbar and Shahrokhian, 2018)
22.	GCG CCC TCT CAC GTG GCA CTC AGA GTG CCG GAA GTT CTG CGT TAT	DNA	*S. aureus*		10^8^ to 10^1^ CFU/mL	10 CFU/mL	Aptasensor based on the FRET between green carbon quantum dot and gold nanoparticle	(Pebdeni et al., 2020)
23.	ATACCAGCTTATTCAATTAGCAACATGAGGGGGATAGAGGGGGT GGGTTCTCTCGGCT	DNA	*S. aureus*	Targets protein A (surface bound virulence factor	–	10 CFU/mL	Impedimetric biosensor based on the protein A-binding aptamer	(Reich et al., 2017)
24.	–	DNA	*MRSA*	–	10^2^ to 10^8^ CFU/mL	Theoretical value = 2 CFU/mL Visual LOD <100 CFU/mL	Aptasensor swab designed for qualitative and quantitative detection, on contaminated non-absorbable surfaces.	(Raji et al., 2021)
25.	GCAATGGTACGGTACTTCCTC GGCACGTTCTCAGTAGCGCTCGCTGG TCATCCCACA GCTACGTCAAAAGTGCACGCTACTTTGCTAA	DNA	*S. aureus*	–	52 to 5.2× 10^7^ CFU/mL	1 CFU/mL	An ultrasensitive sandwich−type electrochemical aptasensor using silver nanoparticle/titanium carbide nanocomposites	(Hui et al., 2022)
26.	–	–	*S. aureus*	–	7.6 × 10^1^ to 7.6 × 10^7^ CFU/mL	1.09 CFU/mL	Dual-recognition SERS biosensor based on teicoplanin functionalized gold-coated magnet NPs as capture probe and *S.aureus* aptamer functionalized silver coated gold NPs as signal probe	(Qi et al., 2022)
27.	NH_2_- GCG CCC TCT CAC GTG GCA CTC AGA GTG CCG GAA GTT CTG CGT TAT	DNA	*S. aureus*	–	10^2^ to 10^8^ CFU/mL	80 CFU/mL	Aptamer and antibiotic-based dual detection sensor combining vancomycin-copper nanoclusters for the recognition and quantification using fluorescence	(Bagheri Pebdeni et al., 2021)
28	–	DNA	*S. aureus*	–	10 to 10^8^ CFU/mL	3 CFU/mL	Electrochemical aptasensor based on gold/nitrogen-doped carbon nano-onions	(Sohouli et al., 2022)
29.	ATCCATGGGGCGGAGATGAGGGGGAGGAGGGCGGGTACCCGGTTGAT	ssDNA	*Listeria monocytogenes*	–	1.4 × 10^1^ to 1.4 × 10^6^ CFU/mL	4 CFU/mL	Solid-state electrochemiluminescence biosensing based on the quenching effect of ferrocene on ruthenium pyridine	(Chen et al., 2021c)
30.	NH2-ATC CAT GGG GCG GAGATG AGG GGG AGG AGG GCG GGT ACC CGG TTGAT	ssDNA	*Listeria* *monocytogenes*	–	10^1^ to 10^8^ CFU/mL	10 CFU/mL	Paper-based electrodes conjugated with tungsten disulfide nanostructure and aptamer for impedimetric detection	(Mishra et al., 2022)
31.	biotin-ATC CAT GGG GCG GAG ATG AGG GGG AGG AGG GCG GGT ACC CGG TTG AT	DNA	*Listeria* *monocytogenes*	–	1.0 × 10^1^ to 1.0 × 10^5^ CFU/mL	6 CFU/mL	Luminol-functionalized AuNF-labeled aptamer recognition and magnetic separation	(Chen et al., 2021b)
32.	–	–	*Listeria monocytogenes*	–	10^2^ to 2 × 10^8^ CFU/mL	2.8 × 10^2^ CFU/mL	Sandwich fluorometric method for dual-role recognition was developed based on antibiotic-affinity strategy and fluorescence quenching effect	(Li et al., 2022)
33.	biotin-TAC TAT CGC GGA GAC AGC GCG GGA GGC ACC GGG GA	DNA	*Listeria innocua*	–	–	1.6 × 10^3^ CFU/mL	Acoustic aptasensor	(Oravczová et al., 2020)
34.	TATCCATGGGGCGGAGATGAGGGGGAGGAGGGCGGGTACCCGGTTGAT	DNA	*Listeria monocytogenes*	–	4.6 × 10^2^ to 4.6 × 10^7^ CFUmL^-1^in pure culture and 6.1 × 10^3^ to 6.1 × 10^7^ CFU/g in spiked fresh lettuce	4.6 × 10^2^ CFUmL^-1^ in pure culture and 6.1 × 10^3^ CFU/g in spiked fresh lettuce	Competitive enzyme-linked aptasensor with rolling circle amplification (ELARCA) assay for colorimetric detection	(Zhan et al., 2020)
35.	TACTATCGCGGAGACAGCGCGGGAGGCACCGGGGA	–	*Listeria monocytogenes*	–	1.4 × 10^1^ to 1.4 × 10^7^ CFUmL	0.88 CFU/mL	Dual recognition and highly sensitive detection by fluorescence enhancement strategy	(Du et al., 2022)
36.	NH2(CH2)6GGGAGCTCAGAATAAACGCTCAA TACTATCGCGGGACAGCGC GGGAGGCACCGGGGATTCGACATGAGGCCCGGATC	DNA	*Listeria monocytogenes*	–	68 to 68 × 10^6^ CFU/mL	8 CFU/mL	Fluorescence aptasensor	(Liu et al., 2021)
37.	NH2-C6-CCC CCG TTG CTT TCG CTT TTC CTT TCG CTT TTG TTC GTT TCG TCC CTG CTT CCT TTC TTG	DNA	*Pseudomonas aeruginosa*	–	3.1 × 10^2^ to 3.1 × 10^7^ CFU/mL	100 CFU/mL	Low-field magnetic resonance imaging aptasensor for the rapid and visual sensing	(Jia et al., 2021)
38.	NH2- CCC CCG TTG CTT TCG CTT TTC CTT TCG CTT TTG TTC GTT TCG TCC CTG CTT CCT TTC TTG	DNA	*P. aeruginosa*	Whole cell	10^2^ to 10^6^ CFU/mL	50 CFU/mL	A magnetic relaxation switch aptasensor	(Jia et al., 2017)
39.	CCCCCG TTGCTTTCGCTTTTCCTTTCGCT TTTGTTCGTTTC GTCCCTGCTTCCTTTCTTG	ssDNA	*P. aeruginosa*	–	10^8^ to 10^5^ CFU/mL	10^5^ CFU/mLfor colour change by the naked eye and 10^4^ CFU/mL for UV–Vis spectrometry	Colorimetric detection by aptamer−functionalized gold nanoparticles	(Schmitz et al., 2023)
40.	GCA-ATG-GTA-CGG-TAC-TTC-CCG-GGG-CCC-GCT-TCT-GGT-GCG-GTG -TAC-TAG-TGA-CCG-CAA-AAG-TGC-ACG-CTA-CTT-TGC-TAA-(CH_2_)6-SH	DNA	*P. aeruginosa*	3-O-C12-HSL (Quorum-Sensing Molecule)	0.5 to 30 µM	0.5 µM	Label-free electrochemical aptasensor for the detection of the 3-O-C12-HSL	(Capatina et al., 2022)
41.	CCC CCG TTG CTT TCG CTT TTC CTT TCG CTT TTG TTC GTT TCG TCC CTG CTT CCT TTC TTG	DNA	*P. aeruginosa*	–	1.28 × 10^3^ to 2.00 × 10^7^ CFU/mL	100 CFU/mL	Graphene oxide quantum dots assisted construction of fluorescent aptasensor	(Gao et al., 2018)
42.	NH2-CCC CCG TTG CTT TCG CTT TTC CTT TCG CTT TTG TTC GTT TCG TCC CTG CTT CCT TTC TTG	DNA	*P. aeruginosa*	–	10^2^ to 10^7^ CFU/mL	33 CFU/mL	Impedimetric aptasensor by using a glassy carbon electrode modified with silver nanoparticles	(Roushani et al., 2019)
Virus								
43.	AGC GGA TCC GAT GGG TGG GGG GGT GGG TAG GAT CCG CG	ssDNA	DENV	Non-structural protein 1	–	2.51 nM in buffer and 8.13 nM in serum	G-quadruplex (GQ)-based fluorescent aptasensor using one-shot detection of NS1	(Mok et al., 2021)
44.	HS_TAGGCAGTGTGGACGAGAGGGAGCTGTCCTGAGAGAGGCCTG TCAACCAGGGGTACCACAACCGAGGGCATA_SH	DNA	DENV-2	E-protein	–	100 infectious units per mL	Porous Au-seeded Ag nanorod networks conjugated with DNA aptamers for impedimetric sensing	(Kumar De et al., 2021)
45.	–	DNA	DENV	surface envelope proteins	10^–6^ to 10^6^ TCID_50 /_mL	1.74 × 10*^-^* ^7^ TCID_50_/mL	AC-electrothermal flow-based rapid biosensor	(Park et al., 2023)
46.	HS(CH_2_)_6_ – TTTTT – ACTAGGTTGCAGGGGACTGCTCGGGATTGCG GATCAACCTAGTTGCTTCTCTCGTATGAT	DNA	DENV-1 and DENV-4	NS1	10 pg to 1 μg/mL	22 pg/mL	Electrochemical aptasensor	(Bachour Junior et al., 2021)
47.	–	DNA	Hepatitis C virus (HCV)	HCV core protein	10 to 70 pg/mL and 70 to 400 pg/mL	3.3 pg/mL	Electrochemical detetction using GQD nanocomposite	(Bachour Junior et al., 2021)
48.	GCGGATCCAGACTGGTGTGCCGTATCCCT CCCTTGTAATTATTTG TTCCATCCGTTCCGCCCTAAAGACAAGCTTC	ssDNA	HCV	HCV core protein	10^− 14^ to 10^−18^ M	15.6 aM	Attomolar detection powered by molecular antenna-like effect in a graphene field-effect aptasensor	(Palacio et al., 2023)
49.	CACAGCGAACAGCGGCGGACATAATAGTGCTTACTACGAC	DNA	Hepatitis B virus (HBV)	HBsAg	–	0.05ng/mL	Chemiluminescent aptasensor based on rapid magnetic separation and double-functionalized gold nanoparticle	(Xi et al., 2018)
50.	NH_2_- TTGGGGTTATTTGGGAGGGCGGGGGTT	DNA	Influenza A virus	H5N1 IAV hemagglutinin	0.2 to 12 ng/mL	114.7 pg/mL	FRET Aptasensors	(Zhao et al., 2021)
51.	GTG TGC ATG GAT AGC ACG TAA CGG TGT AGT AGA TAC GTG CGG GTA GGA AGA AAG GGA AAT AGT TGT CCT GTT G	DNA	H5N1 AIV	–	–	0.0128 hemagglutinin units (HAU)	An Impedance Aptasensor with Microfluidic Chips	(Lum et al., 2015)
52.	Cy3/GGG TTT GGG TTG GGT TGG GTT TTT GGG TTT GGG TTG GGT TGG GAA AAA	DNA	Influenza A/H1N1 virus	–	–	97 PFU/mL	SERS imaging-based aptasensor	(Chen et al., 2020)
53.	Apt 1 H2N-GCT AGC GAA TTC CGT ACG AAG GGC GAA TTC CAC ATT GGG CTG CAG CCC GGG GGA TCC Apt 2 H2N-GTC TGT AGT AGG GAG GAT GGT CCG GGG CCC CGA GAC GAC GTT ATC AGG C Apt 3 H2N-CGT ACG GAA TTC GCT AGC ACG GGG CTT AAG GAA TAC AGA TGT ACT ACC GAG CTC ATG AGG ATC CGA GCT CCA CGT G Apt 4 H2N-CGT ACG GAA TTC GCT AGC CGA CGG TCA ATG CTC GTG AGC CAG TAC ACA CAA TAT ATG TGG ATC CGA GCT CCA CGT G	DNA	Norovirus	NoV capsid protein	–	70 aM	An Aptamer-aptamer Sandwich Assay with Nanorod-enhanced SPR for Attomolar Concentration	(Kim et al., 2018b)
54.	AGT ATA CGT ATT ACC TGC AGC CCA TGT TTT GTA GGT GTA ATA GGT CAT GTT AGG GTT TCT GCG ATA TCT CGG AGA TCT TGC	DNA	Norovirus	–	13 ng/mL to 13 μg/mL	4.4 ng/mL and 3.3 ngmL for MWCNT or GO respectively	Aptamer-based fluorometric determination using a paper-based microfluidic device	(Weng and Neethirajan, 2017)
55.	GCTAGCGAATTCCGTACGAAGGGCGAATTCCACATTGGGCT GCAGCCCGGGG GATCC	DNA	Norovirus	MNV virion	–	200 viruses/mL	Ultrasensitive colorimetric detection using NanoZyme aptasensor	(Weerathunge et al., 2019)
56.	CAG CAC CGA CCT TGT GCT TTG GGA GTG CTG GTC CAA GGG CGT TAA TGG ACA	DNA	SARS-CoV-2-RBD	–	0.5–250 ng/mL	32 ng/mL	Highly sensitive aptasensor using aptamer-gated methylene blue@mesoporous silica film/laser engraved graphene electrode	(Amouzadeh Tabrizi and Acedo, 2022)
57.	–	DNA	SARS-CoV-2	Nucleocapsid protein	–	0.77 to 1.94 ngmL	Fluorescent nanodiamond-based spin-enhanced lateral flow immunoassay and spike protein from different variants	(Wei-Wen Hsiao et al., 2022)
58.	Apt 1 biotin-GCT GGA TGT CAC CGG ATT GTC GGA CAT CGG ATT GTC TGA GTC ATA TGA CAC ATC CAG C Apt 2 biotin-GCT GGA TGT TGA CCT TTA CAG ATC GGA TTC TGT GGG GCG TTA AAC TGA CAC ATC CAG C	DNA	SARS-CoV2	Nucleocapsid protein	–	33.28 pg/mL	Aptamer/antibody sandwich method	(Ge et al., 2022)
59.	GCA ATG GTA CGG TAC TTC CGG ATG CGG AAA CTG GCT AAT TGG TGA GGC TGG GGC GGT	DNA	SARS-CoV-2	–	1 fM to 100 pM	0.389 fM	Aptasensing nucleocapsid protein on nanodiamond assembled gold interdigitated electrodes for impedimetric assessment	(Ramanathan et al., 2022)
60.	TGA CAC CGT ACC TGC TCT-N40-AAG CAC GCC AGG GAC TAT	DNA	Zika virus	–	100 pM to 10 μM	38.14 pM	Electrical biosensor	(Park et al., 2022)
61.	CTTCTGCCCGCCTCCTTCC-(39N)-GGAGACGAGATAGGCGGACACT	DNA	Zika virus	NS1 protein	0.01 to 1000 pg/mL	0.01 pg/mL	Aptasensor based on graphene FETs	(Almeida et al., 2022)

### 3.1 Optical aptasensors

The components of an optical biosensor are an optical transducer system coupled with a biorecognition sensor. Optical biosensors are designed to generate a signal that is directly proportional to the concentration of the analyte (Damborský et al., 2016). Optical aptasensors are biosensors in which the biorecognition sensing element is an aptamer. The transduction method can be SPR, fluorescence, surface enhanced raman scattering (SERS) and chemiluminescence (Uniyal et al., 2023). Optical sensors are frequently used in aptasensors because of their high sensitivity, robustness, reliability, good temporal and spatial control, selectivity, simplicity, versatility, and wide linear range for biomolecule detection (Chen et al., 2021c).

#### 3.1.1 Surface plasmon resonance based aptasensors

When a plane polarized light falls on a thin sheet of metal, plasmons (group of electrons that undergo oscillation due to energy absorption) are formed. In context of aptasensors, aptamer-functionalized metal particles are used. When the analyte binds to the aptamer, it causes changes in the refractive index at the interface, altering the resonance condition of the surface plasmons. These changes can be observed as variations in the angle or intensity of reflected light (Schasfoort, 2017). The sensitivity and selectivity of SPR-based sensors can be significantly improved by utilizing gold nanoparticles linked to ligands that are specific to the target. SPR assays are commonly used in dual-recognition biosensors and sandwich assays to enhance detection capabilities (Kim et al., 2018a).

#### 3.1.2 Fluorescence based aptasensors

In this type of biosensing there are usually two probes involved- the capture probe that binds to the infectious agent and the signaling probe which is usually a nanoparticle that is tagged with a fluorophore. The interaction between the analyte and the aptamers leads to a rise in the fluorescence signal, which is detectable and can be analyzed in both qualitatively and quantitatively. Examples of fluorescent labels are Lanthanide-doped upconversion nanoparticles (UCNPs), silver nanoclusters (AgNCs) (Zhang et al., 2020), carbon quantum dots (CQDs) (Pebdeni et al., 2020), CdTe quantum dots and thiazole orange (Pang et al., 2015).

UCNPs have distinctive optical and chemical characteristics, including excellent photostability, low light scattering, low autofluorescence backgrounds, and low toxicity (Liu et al., 2021). AgNCs have the advantages of high quantum yield, strong photostability, low toxicity, adjustable fluorescence emission, and excellent biocompatibility (Zhang et al., 2020).

An important application of fluorescence spectroscopy is förster resonance energy transfer (FRET). It involves non-radiative transfer of energy from an excited donor fluorophore to an acceptor fluorophore that are in proximity. This phenomenon is also called quenching. Graphene oxide is a commonly used quencher molecule (Verma et al., 2023). For example, Pebdeni et al. discovered that CDQs emit blue-colored fluorescence, which is quenched in the presence of aptamers and gold nanoparticles. With the introduction of specific bacteria, the aptamer-target complex was effectively assembled, leading to the restoration of free CQD emission. The linear range of this aptasensor was 10^8^ to 10^1^ CFU/mL, with a detection limit as low as 10 CFU/mL for *S. aureus* (Pebdeni et al., 2020). Colorimetric aptasensors work by detecting changes in the color due to the binding of the aptamer to the analyte. This is done with the help of UV-visible spectroscopy. A peak is obtained at a specific wavelength and stokes shift takes place (Weerathunge et al., 2019).

#### 3.1.3 SERS based aptasensors

Surface-enhanced Raman scattering (SERS) is a phenomenon in which the Raman scattering signals are amplified by enhancing the sensor surface. Nanostructured surfaces, usually made of metals such as gold or silver, are shaped into nanoparticles, nanorods, or nanostars. These structures demonstrate strong localized surface plasmon resonance (LSPR), resulting in the enhancement of Raman signals of nearby molecules through electromagnetic and chemical mechanisms. The SERS substrates are aptamers and when the infectious agent binds to the aptamer, there is a change in the raman signal that is detected (Zhou et al., 2020).

#### 3.1.4 Chemiluminescence based aptasensors

Chemiluminescence-based aptasensors rely on the emission of light resulting from a chemical reaction between a luminophore (a molecule capable of emitting light) and a substrate or analyte, often facilitated by enzymatic reactions (Chen et al., 2021b). A DNA aptasensor to detect norovirus GII capsid was developed based on guanine chemiluminescence detection and the principle of intra chemiluminescent resonance transfer. The high-energy intermediates formed from the reaction of extra guanines and TMPG transferred the energy to 6-FAM which caused bright chemiluminescence (Kim et al., 2018a).

### 3.2 Electrochemical biosensors

A variety of electrochemical transducer systems, including impedimetric, potentiometric, amperometric, voltammetric, conductometric, and FET-based biosensors, can be integrated with aptamers for enhanced functionality.

#### 3.2.1 Impedimetric aptasensor

When the target analyte binds to the aptamer functionalized sensor surface, inducing changes in the electrical properties at the interface such as charge transfer kinetics, dielectric properties, or surface conductivity at the sensor interface. The change in impedance is converted into a measurable electrical signal. Impedance spectroscopy measures the impedance change of the sensor due to exposure to the target analyte and computes how the sensors electrical impedance changes over a range of frequencies. In a study conducted by Roushani et al. (2019) NH_2_-aptamer was immobilized covalently on the surface of a glassy carbon electrode through electrodeposition modification of AgNPs. The conductivity and the charge transfer resistance before and after the addition of *P.aeruginosa* to the aptasensor was studied. The impedance increases on going from 10^2^ to 10^7^ CFU/mL concentrations of *P. aeruginosa*, and the detection limit was found to be 33 CFU/mL (for S/N=3). In a study conducted by Ramanathan et al. (2022) carbon nanodiamond enhanced gold interdigitated electrode was used to detect the nucleocapsid protein of SARS-CoV-2. The aptasensor which was portable, showed a good selectivity with a lower detection limit of 0.389 fM; at a linear detection range from 1 fM to 100 pM; showing 30 & 33% loss with stability & reusability. A rapid (30 mins) label-free aptasensor was constructed by Bagheryan et al., using screen-printed electrodes (SPEs) that were modified with diazonium salt for the detection of *Salmonella typhimurium* in spiked apple juice samples. The aptasensor had a linear detection range of 1×10^1^ to 1×10^8^ CFU mL^−1^ (Bagheryan et al., 2016).

#### 3.2.2 Voltammetry based aptasensors

In a recent study, Fathi et al. (2020) developed a novel voltammetric aptasensor for detecting *Salmonella enterica* serovar. The sensor utilized a pencil graphite electrode modified with chitosan-coated electrospun carbon nanofibers and gold nanoparticles. The presence of the analyte on the electrode surface led to an increase in charge transfer resistance, with the change in current being measured as a function of voltage. Electrochemical detection of *Salmonella* was achieved using differential pulse voltammetry in a methylene blue solution. The aptasensor demonstrated a linear detection range of 10 to 10^5^ CFU/mL, with a limit of detection (LOD) of 1.223 CFU/mL, outperforming the PCR technique.

#### 3.2.3 Graphene FET based aptasensors

An aptamer with high affinity against HCV (hepatitis C virus) was functionalized on graphene solution-gated field-effect transistors (g-SGFET) and the developed aptasensor was used to amplify and detect the change in conductance caused by the interaction between the aptamer and the HCV core protein (Palacio et al., 2023). Similarly, Almeida et al., fabricated a graphene FET aptasensor to detect Zika virus (ZIKV). The aptamer (termed ZIK60), selected by CE-SELEX was complimentary to the Zika virus non-structural protein 1 (NS1) and counterselection against the NS1 proteins of DENV (serotypes 1, 2, 3, and 4) and YFV (Almeida et al., 2022).

#### 3.2.4 Quartz crystal microbalance based aptasensors

QCM aptasensor is an acoustic (mass-based) piezoelectric biosensor that detect changes in mass on the aptamer immobilized surface of quartz crystal due to its interaction with the analyte molecules by detecting changes in the resonance frequency of the crystal. QCM-based aptasensors are highly sensitive, label free, portable and can be miniaturised and hence are suitable for point-of-care diagnostics. Aptamer selected using whole cell SELEX was utilized to fabricate a QCM sensor to detect *E. coli O157:H7*. The aptasensor had a LOD that was as low as 1.46 × 10^3^ CFU/mL and outperformed most QCM-based immunosensors for pathogen detection. In addition, the quick response time of 50 min showed the possibility of using this aptamer in various other types of biosensors used for rapid detection and investigation of *E. coli* O157:H7 outbreaks (Yu et al., 2018). An interesting study conducted by Wang et al., demonstrates the use of QCM based SELEX to effectively select the ssDNA aptamer and subsequent construction of QCM based aptasensor which was able to detect 10^3^ CFU/mL of *S. typhimurium* within 1 h (Wang et al., 2017a). Another example is a QCM aptasensor in which a nanowell based electrode effectively increased the immobilization capacity of aptamers for the detection of avian influenza virus. The result showed that the binding of target AIV H5N1 onto the immobilized aptamers decreased the sensor’s resonant frequency, and the frequency change correlated to the virus titer. The detection range of 2^−4^ to 2^4^ hemagglutination units (HAUs)/50 μL was obtained with a detection limit of 2^−4^ HAU/50 μL for AIV H5N1 with a detection time of 10 mins using a label free assay. (Wang et al., 2017a, 2017b)

### 3.3 Dual recognition aptasensor

As the name suggests, dual recognition sensors make use of two different recognition principles facilitating a highly specific detection. Li et al., developed an aptasensor for detecting *S. typhimurium* by combining the methods of immune hybridization chain reaction (HCR) with SERS achieving double amplification and high sensitivity with a limit of detection of 6 CFU/mL in 3.5 h (Li et al., 2021). Bagheri Pebdeni et al., proposed an aptamer and antibiotic-based dual detection sensor that combines copper nanoclusters (CuNCs) as an effective approach for the recognition and quantification of *S. aureus*. The use of dual receptors enhanced fluorescence signal linearly with *S. aureus* concentrations between 10^2^ -10^8^ CFU/mL, and the detection limit was 80 CFU/mL after 45 min (Bagheri Pebdeni et al., 2021). Aptasensors like the electrochemiluminescence aptasensors come under both electrochemical and optical sensors. It works by detecting the luminescence that is produced due to the electrochemical interactions between the aptamer and the analyte molecules (Chen et al., 2021c; Chen et al., 2021b).

### 3.4 Sandwich assay based aptasensors

A sandwich assay involves two aptamers – the capture probe and the signal probe. The capture probe is immobilized on the surface of the sensor and after the analyte is added, the signal probe is added forming an aptamer-aptamer sandwich platform. This method is desirable because of the high sensitivity and selectivity that it offers. S. Kim et al., demonstrated a nanorod enhanced SPR with sandwich enzyme-linked immunosorbent assay (ELISA) for the attomolar detection of the norovirus (NoV) capsid protein (Kim et al., 2018b). RNA aptamer-based sandwich assays were used to detect the NS1 protein of dengue virus serotype 2 and a LOD of 2 nM was attained (Thevendran et al., 2023).

Another notable example is an aptamer/antibody sandwich constructed by Ge et al., for the digital detection of SARS-CoV2 nucleocapsid protein using fluorometry. The detection limit of this digital method for N protein was 33.28 pg/mL, which was 300 times lower than traditional double-antibody sandwich-based ELISA (Ge et al., 2022). Even though sandwich ELISA assay offers various advantages, it has a complex workflow, more optimization is required, is labor intensive and the time of detection is a little high.

### 3.5 Other aptasensors

There are aptasensors based on principles other than the above mentioned, for example, F. Jia et al. developed a low-field magnetic resonance imaging (LF-MRI) aptasensor based on the difference in magnetic behavior of two magnetic nanoparticles covalently immobilized with aptamers for the rapid detection of *P.aeruginosa*. Under optimum conditions, the LF-MRI platform provides both image analysis and quantitative detection of *P. aeruginosa*, with a detection limit of 100 CFU/mL (Jia et al., 2021).

Aptamer-based assays represent a significant advancement in the diagnostics of infectious diseases, addressing the limitations of traditional methods. Optical aptasensors, including surface plasmon resonance, fluorescence, and surface-enhanced Raman scattering, excel in sensitivity and specificity, ideal for detailed biomolecule detection. Electrochemical aptasensors, such as impedimetric, voltammetric, and graphene FET-based sensors, offer robust, portable solutions with high sensitivity for point-of-care applications. Meanwhile, dual-recognition and sandwich assay-based aptasensors combine multiple detection principles to enhance accuracy and detection limits. This comprehensive range of aptamer-based technologies demonstrates their potential to revolutionize diagnostic practices by providing versatile, efficient, and precise tools for infectious disease management. The most suitable method can be selected by understanding the strengths and limitations for each approach.

## 4 Notable aptasensor case studies

### 4.1 For the detection of methicillin resistant *Staphylococcus aureus-contaminated* surfaces

Methicillin-resistant Staphylococcus aureus (MRSA) is a well-known pathogen that causes healthcare-associated infections. Hospitals with contaminated environments are important sources for the spread of MRSA and other nosocomial infections. In a study, researchers have developed a new swab called a pathogen aptasensor which can specifically detect MRSA on contaminated non-absorbable surfaces. The visual detection limit of the MRSA aptasensor swab was less than 100 CFU/mL, and theoretically, using a standard curve, it was 2 CFU/mL. The assay has a short turnaround time of 5 minutes, with a linear range of quantitation from 10^2 to 10^5 CFU/mL. The MRSA aptamers bind to the swab’s activated aldehyde group, and when exposed to an MRSA-contaminated surface, the activated nanobeads conjugate with the aptamer, causing the swab to turn blue. The intensity of the color change is proportional to the concentration of MRSA, allowing for both qualitative and quantitative detection (Raji et al., 2021).

### 4.2 Simultaneous detection of *E.coli* O157:H7 and *S.typhimurium*

Simultaneous detection of *E.coli* and *S.typhimurium* was achieved using an evanescent wave dual-color fluorescence aptasensor based on time resolved effect. Two fluorescence labeled aptasensors, Cy3-apt-E and Cy5.5-apt-S that were complimentary to *E.coli* O157:H7 and *S.typhimurium* were alternatively excited by evanescent waves originated from 520 nm to 635 nm excitation lights, respectively. The fiber nanoprobe with *in-situ* etched nanopores was used for distinguishing free aptamer and aptamers bound to pathogenic bacteria based on the limited penetrated depth of evanescent wave and the significant size difference of bacteria and nanopore. The *E. coli* O157:H7 and *S. typhimurium* were directly and simultaneously quantitated in less than 35 min without the requirement of the complex immobilization of biorecognition molecules and bacteria enrichment/separation processes. The limits of detection of *E. coli* O157:H7 and *S. typhimurium* were 340 CFU/mL and 180 CFU/mL, respectively (Fang et al., 2021).

### 4.3 Colorimetric aptasensor for detecting *Salmonella* spp., *Listeria monocytogenes*, and *Escherichia coli* in meat samples

Aptasensors are revolutionizing infectious disease detection by enhancing the specificity and sensitivity of aptamers. These biosensors provide versatile solutions for streamlining diagnostic processes in healthcare by rapidly and precisely identifying pathogens.

A recent study introduced a quick detection method that can simultaneously identify *Salmonella* spp., *Listeria monocytogenes*, and *E. coli*. This method uses visual colorimetric detection with labeled colloidal gold nanoparticles and UV absorbance determination at optimized wavelengths of 625 nm and 525 nm. The aptasensor has a detection limit as low as 10^5^ CFU/mL. Notably, this colorimetric aptasensor enables one-step detection without the need for pre-culture, DNA extraction, or amplification steps. As a result, it provides a simple, rapid, specific, and qualitative assay suitable for point-of-care testing, allowing for direct detection of multiple foodborne pathogens (Ledlod et al., 2020). Additionally, exploring virulence factors as potential targets for aptamers is helping us understand pathogen behavior and leading to the development of targeted therapeutic interventions.

## 5 Aptamer applications: targeting virulence factors and recent advances

### 5.1 Virulence factors and potential aptamer targets

**Figure 3:**
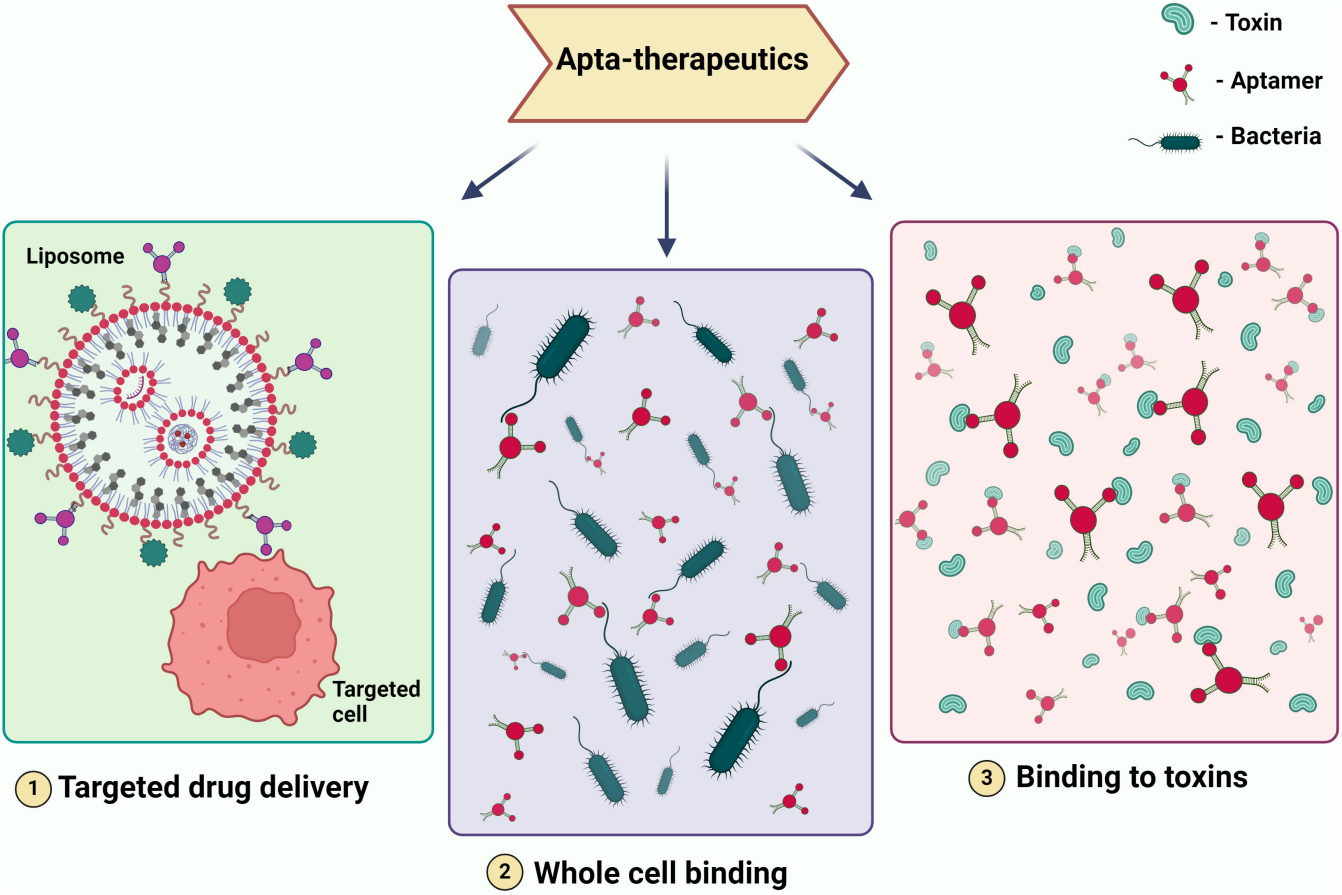
Illustrating Therapeutic Modalities Employing Aptamers. The figure depicts various therapeutic applications of aptamers, including (1) targeted drug delivery, where aptamers are used to direct drugs specifically to diseased cells; (2) whole cell binding, where aptamers bind to specific cells for therapeutic purposes; and (3)binding to toxins, where aptamers neutralize toxins by binding to them. These modalities showcase the potential of aptamers in precision medicine and targeted therapies.

Aptamer is one of the most promising therapeutic candidates because of its selectivity. In the field of therapeutics, they serve various crucial roles, including acting as a drug delivery vehicle (Ninomiya et al., 2014), functioning as a targeting molecule for genes or whole cells, thereby reducing the expression of virulent genes in pathogens and enhancing susceptibility to the immune system (Lai et al., 2014). Furthermore, it serves as a binding agent for toxins and specific proteins that contribute to increased pathogen virulence (Gribanyov et al., 2021). When it comes to treating viral infections that have no known treatment and drug-resistant microorganisms that cause infectious diseases, aptamers may be a useful therapeutic tool (**Figure 3**).

#### 5.1.1 Bacteria

By removing important virulence components from bacteria, aptamers present a viable strategy for treating bacterial illnesses. (**Tables 2**, **3**). The innovative technology enhance the treatment efficacy against pathogens such as *Staphylococcus aureus*, *Mycobacterium tuberculosis*, *Salmonella typhi*, *Listeria monocytogenes*, *Streptococcus pneumoniae*, and *Escherichia coli*. In the fight against *S. aureus* infections, aptamers AT-33 and AT-36 have been specifically engineered to target and neutralize the α-toxin, a key virulence factor. These aptamers effectively inhibit α-toxin-induced cell death and cytokine upregulation in human cells, offering a promising therapeutic approach (Ommen et al., 2022). Another set of aptamers targets *S. aureus* biofilms, binding to the biofilm matrix to enhance antibiotic delivery and significantly improve treatment outcomes by overcoming biofilm-associated resistance. This dual approach of targeting both toxins and biofilms represent a significant advancement in therapeutic strategies against *S. aureus* infections.

**Table 2:** Therapeutic techniques and mechanisms of aptamers against bacteria.

Target	Conjugated With	Target site	Mechanism	Reference
*Salmonella* species				
*S. choleraesuis*	Ampicillin	Flagella	▪ Aptamer 3 targets flagella, causing loss of bacterial motility decreasing adherence to the matrix surface, and reinforces hydrodynamic and repulsive forces which inhibit biofilm formation. ▪ Aptamer 3 may also serve as an antibiotic carrier helping ampicillin to penetrate biofilms to eradicate bacteria and to overcome biofilm tolerance to drugs	(Lijuan et al., 2017)
*S. enteritidis*	–	Sip A protein (SPI –*Salmonella* pathogenicity island)	▪ Apt17, an aptamer targeting SipA an effector protein secreted by Type Three Secretion System (T3SS). ▪ It facilitates the invasion of *Salmonella* cells by triggering membrane ruffling	(Shatila et al., 2020b)
*S. Typhimurium and S. Enteritidis*	–	Sip A protein (SPI –*Salmonella* pathogenicity island)	▪ Targeting *Salmonella* invasion protein (SipA) ▪ A type three secretory system effector protein blocking this helps in anti-adhesion and anti-invasion property against *Salmonella Enteritidis*	(Shatila et al., 2020a)
*S. Typhimurium and S. Enteritidis*	Using rolling circle amplification	-	▪ Use complementary sequences of recently described (anti-ST and anti-SE) DNA aptamers as a template to develop RCA-p. ▪ The use of RCP-p is done to increase the bacteriostatic effect on the bacteria	(Hameed et al., 2022)
*Salmonella enterica serovar typhimurium*	Gold nano particles	Membrane disruption, Intracellular interaction.	▪ Involve the binding of the AMPs to lipopolysaccharide and lipoteichoic acid ▪ With subsequent membrane disruption through pore formation or other processes ▪ AMPs are drugs delivered by aptamer nanoparticle complex	(Yeom et al., 2016)
*Salmonella enterica Serovar typhi*	–	Preferentially bind type IVB pilli	▪ RNA Aptamer is used to bind to IVB pilus operon and stops IVB PILUS formation ▪ Which helps *S.enterica serovar typhi* to attach to cells which increases its pathogenicity	(Pan et al., 2005)
*Staphylococcus aureus*				
*S aureus*	Teicoplanin and PLGA nanoparticles	D-Ala, D-Ala site in peptidoglycan	▪ Aptamer is used to bind to the bacteria and is conjugated with teicoplanin encapsulated in PLGA ▪ Which stops the cell wall synthesis by blocking the D-Ala, D-Ala site	(Ucak et al., 2020)
*S. aureus*	–	Alpha toxin and transcriptional activators of *TNF-alpha* and *IL 17* gene	▪ Aptamers are specific to their targets through SELEX process, so they bind directly to the alpha toxin	(Vivekananda et al., 2014)
*S .aureus* *(MRSA)*	Magnetic graphene oxide	Whole-cell	▪ The conjugated magnetic graphene oxide (MGO) benefits from the aptamer ▪ When it is exposed to NIR light, it produces heat that aids in the death of MRSA.	(A Ocsoy et al., 2021)
*S aureus (MRSA)*	Anti-galactosyltransferase (anti-α-gal)	Whole-cell and help immune system enhance the fight against *MRSA*	▪ Anti-galactosyltransferase, or anti-α-gal, is conjugated with MRSA specific aptamer. ▪ Anti-α-gal alerts the immune system to the presence of bacteria and aids in its prompt elimination.	(Doherty et al., 2023)
*S aureus*	NaY0.28F4:Yb0.70, Er0.02 magnetic nanoparticle	As of now no targets but This can be used as a potential therapeutic	▪ The magnetic nanoparticle NaY0.28F4:Yb0.70, Er0.02 coupled with aptamer is utilized to draw all of the bacterial cells into one location, offering it a possible therapeutic use.	(Duan et al., 2012)
*Mycobacterium tuberculosis*				
*Mycobacterium tuberculosis*	–	Malate Synthase	▪ Aptamer attaches itself to the Mtb's malate synthase, ▪ Inhibiting adhesin function and preventing bacterial invasion.	(Dhiman et al., 2019)
*Mycobacterium tuberculosis*	–	HupB protein	▪ Aptamer attaches itself to the HupB protein and prevents it from working.	(Kalra et al., 2018)
*Mycobacterium tuberculosis*	Biotin	DevR dimer	▪ The DevR dimer becomes dysfunctional when an aptamer attaches to it, inhibiting transcription.	(Chauhan et al., 2022)
*Listeria monocytogenes*				
*Listeria monocytogenes*	Porous silica Nanoparticles	Whole-cell	▪ The aptamer is coupled to porous silica nanoparticles and loaded with benzalkonium chloride (BAC). ▪ Because BAC is toxic, it cannot be utilized in treatment; instead, an appropriate antibiotic can be employed to effectively target the bacteria.	(Sudagidan et al., 2021)
*Listeria monocytogenes*	Antibody of *L. monocytogenes*	Antigen	▪ When an aptamer and bacterial antibodies are conjugated The antigen found in the bacterium may be targeted therapeutically.	(Du et al., 2022)
*Listeria monocytogenes*	Bacteriocin (nisin with leucocin F10)	Cell membrane	▪ Aptamer in connection with Nisin and Leucocin F10 Once it attaches to bacteria, the aptamer pores the bacteria's surface.	(Turgis et al)
*Streptococcus pnuemoniae*				
*Streptococcus pnuemoniae*	α-Gal epitope	Whole cell	▪ An aptamer is designed to attach to an α-Gal epitope, creating an alphamer that targets bacteria ▪ Initiating opsonization and phagocytosing the pathogen by anti-α-Gal antibody.	(Kristian et al., 2015)
*Streptococcus pnuemoniae*	Graphene oxide (GO)		▪ This aptamer has the potential to be a therapeutic tool when combined with drugs that are specific to this bacteria	(Bayraç and Donmez, 2018)
*Streptococcus pnuemoniae*	–	PavA and FHbp	▪ Researchers created an aptamer that binds to the virulent proteins PavA and FHbp. ▪ When the aptamer binds to PavA, it prevents the bacteria from attaching to fibronectin ▪ When it binds to FHbp, it compromises the ability to evade the immune system and kills the bacteria.	(Escolano et al., 2017)
*Escherichia coli*				
*E. coli*	–	Targets adhesins and colonization factor eg: Afimbrial Adhesins	▪ Aptamers were employed to lower the biofilm activity.	(Kusumawati et al., 2022)
*E.coli*	–	Cell membrane	▪ Aptamer was designed to attach to the elements of the cell membrane. ▪ It is also utilized to prevent from forming biofilms.	(Oroh et al., 2020)
*E.coli*	–	LPS	▪ This paper's researchers have inferred that aptamer attaches to the LPS ▪ This method may be exploited as a treatment option in addition to detection.	(Zou et al., 2018)
*E.coli*	AuNPs and Antimicrobial peptides	Cell membrane	▪ Antimicrobial action is demonstrated by HPA3P, a derivative of HP(2-20) ▪ AMP with substitutions of E9P connected with gold nanoparticles and aptamer pair (AuNPs-Apt).	(Lee et al., 2017)

**Table 3:** Virulence factors for aptamer-based targeting of bacterial pathogens.

Virulent factors	Bacteria						
	*L. monocytogenes*	*S.aureus*	*S.typhi*	*M.tuberculosis*	*E.coli*	*S.pnuemoniae*	Reference
**LPS (Endotoxin)**			**+**		**+**		(Chessa et al., 2014) (Somerville et al., 1999)
**Colonization factor**		**+**(adhesins)			**+**		(Gerlach et al., 2007) (Gerlach et al., 2007)
**Listeriolysin O**	**+**						(Portnoy et al)
**Phospholipases**	**+**	**+**					(Faucher et al., 2008) (Kadurugamuwa and Beveridge, 1995)
**ACT A**	**+**						(Pistor et al., 1994)
**Capsules**	**+**	**+**	**+**	**+**	**+**	**+**	(Bai et al., 2021)
**Exotoxins**	**+**(Listeriolysin O)	**+** (hemolysin, leukotoxin, exfoliative toxin, enterotoxin, and toxic-shock syndrome toxin-1 (TSST-1).)	**+**(typhoid toxin)	**+**(necrotizing toxin)	**+**	**+**(pneuemolysin)	(Portnoy et al) (Otto, 2014) (Fowler and Galán, 2018) (Sun et al., 2015) (Kaper et al., 2004) (Nishimoto et al., 2020)
**ESAT-6**				**+**			(Sreejit et al., 2014)
**Surface adhesins**		**+**	**+**	**+**	**+**	**+**	(Gerlach et al., 2007) (Johnson, 1991)
**Protein A**		**+**					(Palmqvist et al., 2002)
**Salmonella pathogenicity island**			**+**(SPI1,SPI2, SPI3)				(Lerminiaux et al., 2020)
**Vi antigen**			**+**				(Zhang et al., 2022)
**Pneumococcal factors (*psp k , sir A, pmp A*)**						**+**	(Brooks and Mias, 2018)
**Flagella**	**+**		**+**		**+**		(Winter et al., 2009) (Grü ndling et al., 2004)
**Protein Kinases**	**+**				**+**	**+**	(Wang & Koshland, 1978) (Canova and Molle, 2014)
**Mannose capped lipoarabinomannan**				**+**			(Turner and Torrelles)
**Phthiocerol Dimycocerosate**				**+**			(Augenstreich et al., 2020)

To identify and target important molecules connected to M. tuberculosis, aptamers have also been developed. For instance, mannose-capped lipoarabinomannan (ManLAM), a major glycolipid on the bacterial surface, serves as a target for specific aptamers, aiding in both diagnostic and therapeutic applications. Additionally, aptamers targeting the GlcB and HspX antigens disrupt bacterial metabolism and persistence, offering potential therapeutic benefits (Zhou et al., 2021). Furthermore, aptamers targeting ESAT-6, a critical virulence factor secreted by *M. tuberculosis*, can reduce the bacterium’s virulence and increase its vulnerability to immune system attacks (Sreejit et al., 2014). *M. tuberculosis* produces a lipid called phthiocerol dimycocerosate (PDIM), which is essential to the pathogenicity and virulence of the bacteria (Augenstreich et al., 2020).

Researchers have developed an aptamer that interacts with and neutralizes the InvA gene of *S. typhi*, a crucial element in the bacterium’s invasion process. Additionally, the S9 aptamer targets the outer membrane protein of *S. typhi*, further contributing to the bacterium’s neutralization (Pathania et al., 2017; Yang et al., 2013). SPI1, or *Salmonella* pathogenicity island 1, is essential for *Salmonella’s* interaction with host cells, facilitating penetration through the T3SS, also known as the needle complex, which assembles proteins to translocate effector proteins into host cells (Raffatellu et al., 2005; Lerminiaux et al., 2020). The pathogenicity of *S. typhi* is enhanced by the release of typhoid toxin and the Vi capsular antigen, which has anti-opsonic and antiphagocytic properties (Galán, 2016; Tran et al., 2010; Wain et al., 2005). These harmful factors can be targeted by specifically curated aptamers.

In order to prevent L. monocytogenes from invading host cells, aptamers that target InlB, one of the bacteria’s virulence factors, have been created. By blocking this key infection pathway, these aptamers offer a promising therapeutic strategy for preventing *L. monocytogenes* infections (Chen et al., 2024). *L. monocytogenes* produces listeriolysin O (LLO), a pore-forming toxin dependent on cholesterol (Dramsi and Cossart, 2002). LLO damages the vacuolar membrane, facilitating bacterial escape into the cytosol (Petrišič et al., 2021). These vulnerable parts of the pathogen can be exploited by targeting them with protein-specific aptamers.

For *S. pneumoniae*, aptamers have shown good specificity; the Lyd-3 aptamer in particular has shown promise. Lyd-3 effectively inhibits biofilm formation, a critical factor in the pathogen’s virulence and antibiotic resistance. By significantly reducing biofilm formation, Lyd-3 enhances treatment outcomes, especially when used in combination with antibiotics (Afrasiabi et al., 2020). The pneumococcus’s polysaccharide capsule is a significant virulence component, aiding in immune evasion and colonization (Jonsson et al., 1985). PspK mediates adherence to human epithelial cells, independent of the pneumococcal isolate genetic background (Keller et al., 2013).

Four aptamers have demonstrated high affinity and specificity for *E. coli* cells, making them valuable tools for both diagnostic and therapeutic applications. These aptamers offer precise detection and effective targeting of *E. coli* (Marton et al., 2016). *E. coli* causes various infections, including urinary tract infections, and relies on colonization factors and toxins for virulence. Aptamers can target these virulent factors, disrupting *E. coli’s* pathogenic mechanisms and enhancing treatment efficacy (Johnson, 1991; Kaper et al., 2004; Terlizzi et al., 2017).

By leveraging the specificity and affinity of aptamers, we can target key virulence factors in various bacterial pathogens, offering innovative and effective therapeutic strategies.

#### 5.1.2 Virus

Aptamers are also increasingly recognized for their potential to combat viral infections by targeting and neutralizing specific viral components (**Table 4**). It can enhance the efficacy of existing antiviral treatments and provides new therapeutic avenues for diseases like Zika virus, Nipah virus, Ebola virus, and Influenza A virus.

**Table 4:** Therapeutic techniques and mechanisms of aptamers against the virus.

Virulence factors of Viruses					
S. No.	Virus	Virulence factor	Function	Mechanism	Reference
1	Zika	NS1 (non-structural protein 1)	Immune evasion and modulation	▪ NS1 prevents the synthesis of interferon-beta (IFN-β), ▪ Essential for the antiviral immune response.	(Rastogi and Singh, 2020)
		NS2A and NS4B	Viral replication	▪ The Zika virus's NS2A contributes to the suppression of NF-κB promoter activity. ▪ NS2A is composed of a central region (the bridge) that passes through a cellular compartment (ER) & six arms (segments) that extend outward from the central region.	(Lee et al., 2020) (Nutho et al., 2019)
		Envelope protein	Entry into host cell and helps in assembly of new viral particles	▪ E protein promotes the production of viral particles by interacting with apolipoprotein E, a protein involved in lipid metabolism. ▪ C-type lectin receptors in the host cell are involved in receptor-mediated endocytosis.	(Nutho et al., 2019) (Agrelli et al., 2019)
		Capsid protein	Helps in formation of new virus particles	▪ capsid protein forms overall positively charged dimers that bridge RNA and lipid membrane surfaces. ▪ The protein exists as dimers with four α helices and a long pre-α1 loop, contributing to its unique structure	(Shang et al., 2018)
2	Ebola	Viral protein 24 (VP24)	Interferes in host interferon and evades host immune system	▪ VP24 suppresses interferon-dependent signaling, of interferon alpha/beta (IFN-α/β). ▪ The host's antiviral response is interfered, which makes it easier for the virus to multiply and propagate.	(Zhang et al., 2012)
		VP30	Helps in transcription and replication	▪ Dynamic phosphorylation of VP30 occurs at six serine residues at the N-terminus. ▪ This post-translational alteration affects VP30's function in viral transcription and replication by regulating its activity in conjunction with dephosphorylation.	(Lier et al., 2017)
		VP35	Interferes with host interferon regulatory factor (IRFs)	▪ VP35 interacts with the PKA-CREB1 pathway, a set of intracellular chemical signals. ▪ A biological protein known as AKIP1 is bound by VP35, starting a chain reaction. ▪ PKA (Protein Kinase A) and CREB1 (cAMP Response Element-Binding Protein 1), two important participants, are activated by this binding. ▪ Following activation, CREB1 is drawn to viral inclusion bodies, which are particular structures created when the Ebola virus infects a host.	(Zhu et al., 2022)
		VP40	Formation and release of viral particles from infected cells	▪ SUMOylation is a post-translational modification that controls VP40. ▪ Affects the stability, nucleocapsid recruitment, structure, and budding of the virus.	(Baz-Martnez et al., 2016)
		L Polymerase	RNA dependent RNA polymerase involved in replication and transcription	▪ The process includes the polymerase starting RNA synthesis from scratch, or de novo, without the aid of an existing primer.	(Yuan et al., 2022)
3	Nipah	Nucleoprotein (N protein)	responsible for enclosure of viral RNA genome	▪ The N protein facilitates the interchange of N-terminal (NTARM) and C-terminal subdomains (CTARM) and lateral interactions that lead to the creation of a stable homopolymer structure. ▪ This particular structural configuration enhances the nucleocapsid's integrity.	(Ker et al., 2021)
		Phosphoprotein (P protein)	Crucial for viral RNA synthesis and synthesis	▪ Viral polymerase activity and viral RNA synthesis are regulated by overexpression of the Nipah virus nucleocapsid protein (N) ▪ Which indicates the complex interaction between P and other viral components.	(Ranadheera et al., 2018)
		Matrix protein (M protein)	Involved in assembly and budding of new vial particles	▪ The induction of interferon-beta (IFNβ) at the level of the TBK1/IKKϵ kinases is inhibited by the NiV matrix protein.	(Bharaj et al., 2016)
		Fusion protein (F protein)	Helps in viral entry into host cells	▪ The attachment (G) protein and the NiV-F protein work together to mediate viral entry and syncytium formation. ▪ Syncytium formation is the process by which adjacent and infected cells combine to promote the spread of the virus.	(Aguilar et al., 2006)
4	Influenza A virus	Hemagglutinin HA	Helps in adherence to host cell	▪ Low pH inside endosomes causes HA to undergo a conformational shift after attachment. ▪ The fusing of the viral and endosomal membranes can be mediated by HA ▪ Conformational shift exposes a fusion peptide. ▪ The viral genome must pass through this stage in order to enter the cytoplasm of the host cell.	(Brandenburg et al., 2013)
		Neuraminidase NA	Releases viral particles from infected cells	▪ Neuraminidase is an exosialidase that breaks the α-ketosidic bond between the sugar residue next to the sialic acid on the surface of host cells that are infected. ▪ The release of offspring viruses from the host cell membrane depends on this cleavage.	(McAuley et al., 2019)
		PB1, PB2, PA	Viral replication	▪ The catalytic component responsible for RNA-dependent RNA polymerase (RdRP) activity is called PB1. ▪ PB2 participates in the cap-snatching process, responsible for the start of viral transcription. To facilitate the production of viral mRNA, the viral polymerase snatches the 5' cap structure from host pre-mRNAs. ▪ In the cap-snatching procedure, PA is an essential component. Sue to its endonuclease activity, host mRNA can be broken down close to the 5' cap structure. ▪ Afterwards, PB2 uses this cleaved cap to start viral transcription.	(Binh et al., 2013) (Lerminiaux et al., 2020) (Ma et al., 2017)
		NP	Responsible for enclosure of viral RNA genome	▪ The results show that NP serves a variety of purposes throughout the life cycle of the virus, and its requirement varies depending on the particular circumstances or context of the viral activities under investigation.	(Turrell et al., 2013)
		M1 M2	Involved in assembly and budding of new vial particles	▪ M1's conformation may be affected by association with M2, which would promote the elongation of viral budding. ▪ The effective release of new virus particles depends on this interaction.	(Roberts et al., 1998)
		NS1 NS2	Immune evasion	▪ NS1 suppresses host's antiviral defenses by blocking several pathways, including interferon generation and activation of PKR (Protein Kinase R). ▪ The nuclear export of viral ribonucleoprotein (vRNP) complexes is facilitated by NS2. ▪ It controls the movement of NS2 mRNA that has been spliced and its precursor, NS1 mRNA, making it easier for vital viral components to be exported from the nucleus into the cytoplasm.	(Huang et al., 2017) (O’Neill et al., 1998)
5	Noro virus	VP1 (major capsule protein)	Formation of viral capsid, contributes to stability of virion	▪ The capacity of the virus to reproduce in B cells is closely correlated with the projecting domain of VP1 ▪ Potential function for this domain in determining norovirus virulence.	(Zhu et al., 2016)
		VP2 (minor structural protein)	Contributes for the stability of the virion	▪ VP2 experiences coevolution and is the cause of most illnesses. Its significance in the infection process is shown by the coevolutionary dynamics.	(Hong et al., 2022)

Zika virus, a member of the Flaviviridae family transmitted by Aedes aegypti mosquitoes (Diagne et al., 2015), has been targeted with various antiviral strategies. Ribavirin has shown efficacy in suppressing viremia in ZIKV-infected STAT-1-deficient mice (Kamiyama et al., 2017), while favipiravir and BCX4430 inhibit viral RNA synthesis by targeting viral RNA-dependent RNA polymerase (Furuta et al., 2009; Eyer et al., 2017). The similarity between the envelope proteins of dengue and Zika viruses underscores their close evolutionary relationship (Lunardelli et al., 2023). NS1 protein plays critical roles in ZIKV replication (Valente and Moraes, 2019), and aptamer technology holds promise for enhancing antiviral drug efficacy by targeting specific virulence factors (Feng et al., 2011).

Nipah virus lacks specific antiviral treatments, making aptamer-based therapies a potential breakthrough by targeting its virulence factors, such as the F protein that mediates viral entry through ephrin B2/B3 receptors (Sun et al., 2018; Weis and Maisner, 2015). The Nipah virus V protein inhibits STAT proteins, crucial for interferon signaling, enhancing viral pathogenesis (Shaw et al., 2004). Aptamers designed to bind these proteins could mitigate infection severity.

Ebola virus VP35 and VP24 proteins are key virulence factors that disrupt host immune responses (Leung et al., 2010; Zhang et al., 2012), with aptamers identified to target VP35’s interferon inhibitory domain (Binning et al., 2013). These aptamers offer potential therapeutic avenues against Ebola virus by restoring interferon response pathways.

Influenza A viruses, characterized by their surface proteins HA and NA, play crucial roles in viral entry and replication (Bouvier and Palese, 2008). Aptamers targeting HA have demonstrated significant antiviral effects in animal models, inhibiting viral replication and reducing infection rates across different influenza strains (Nobusawa, 1997; Gopinath et al., 2006; Jeon et al., 2004). Aptamer research continues to explore novel therapeutic strategies, addressing the challenges posed by viral mutation and enhancing treatment efficacy (Musafia et al., 2014; Sanjuán, 2012).

### 5.2 Recent advancements and case studies

#### 5.2.1 Notable developments in aptamer research for infectious diseases

##### 5.2.1.1 Gold nanoparticle-DNA aptamer conjugate-assisted delivery of antimicrobial peptide (CA2634987A1)

Gold nanoparticles are a durable and widely used delivery technology that offers various benefits over liposomes and PLGA. It was demonstrated that combining antimicrobial peptides with a gold nanoparticle-aptamer complex was effective in eliminating intracellular Salmonella enterica serovar Typhimurium (Yeom et al., 2016).

##### 5.2.1.2 Point-of-care SARS-CoV-2 salivary antigen testing with an off-the-shelf glucometer (WO2022016163A2)

An innovative test technique that combines a pre-conjugated aptamer with the enzyme invertase, which is then attached to a magnetic bead. Because the aptamer is highly specific to the antigen found in the corona virus, it goes through a confirmational change that releases the enzyme into the medium, where it is separated by magnetic separation. The medium’s invertase then breaks down sucrose into glucose, and measuring the glucose yields an assay of the antigen present in the sample. This model operates on this concept (Singh et al., 2021).

##### 5.2.1.3 Graphene aptasensor for the detection of hepatitis C virus (EP4124855A1)

Changes in their surroundings, particularly the attachment of bio-receptors to the graphene surface, cause Graphene Field-Effect Transistor Biosensors (gFET) to detect changes in electrical metrics, such as conductivity. Graphene’s sensing potential is increased by chemical modification. As an example of how biological molecules can be sensed, researchers have created chemically functionalized gFETs that can detect negatively charged exosomes when they are bound to the graphene surface. In this particular case, researchers have created sgFETs with aptamer that can detect HCV protein even at lower concentrations making it an ultrasensitive aptasensor. Attomolar detection of the viral protein target is made possible by the enhanced sensitivity brought about by induced polarization at the graphene interface (Kwong Hong Tsang et al., 2019; Palacio et al., 2023).

##### 5.2.1.4 Aptamer binding hemagglutinin of H7N7 subtype influenza virus (JP2014008002A)

An aptamer capable of differentiating between influenza A serotypes and interfering with the HA-glycan interaction was created by researchers. To ensure the aptamer’s stability in the presence of endo-ribonucleases, 2′-fluoro cytidine is employed, which does not interfere with its binding to HA. This aptamer has applications in detecting and diagnosing H5N1 and H7N7 viruses, as well as in synthesizing virucidal drugs that selectively target these viruses, impeding their early interactions with hosts (Suenaga and Kumar, 2014). The glycoprotein known as hemaglutinin (HA), which is present on the influenza virus’s surface, is essential to the virus’s capacity to bind to and penetrate host cells. Aptamers work by specifically targeting HA, which stops the virus from attaching to host cell receptors and preventing it from entering the cells (Zou et al., 2019)

#### 5.2.2 Case studies: aptamers that inhibit biofilm

**Figure 4:**
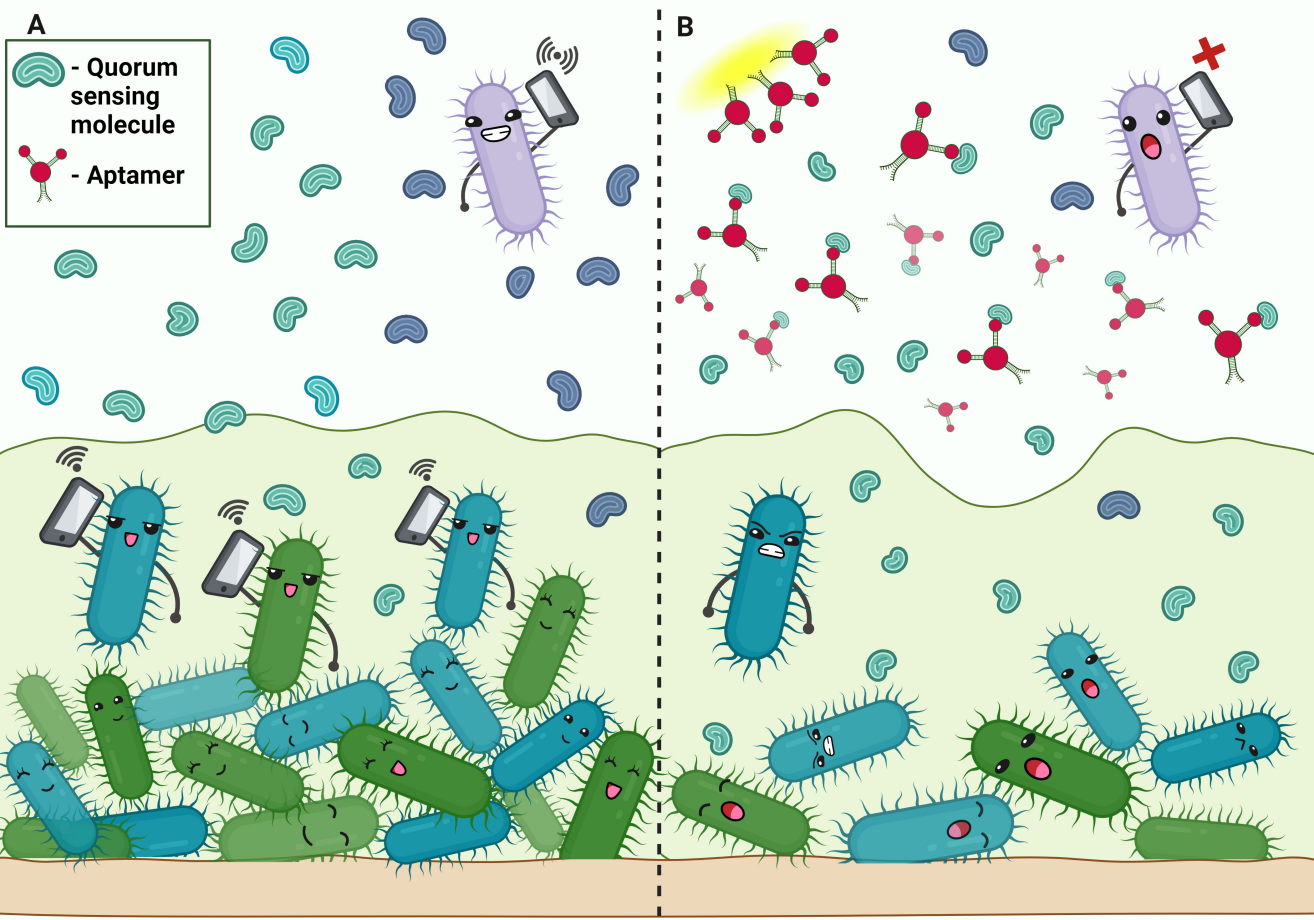
Aptamers disrupting quorum sensing in biofilm. **(A)** Communication in bacterial biofilm by quorum sensing molecule. **(B)** Aptamer binds to the quorum-sensing molecules, disrupting signaling.

Quorum sensing, a mechanism that enables signaling and communication within bacteria, plays a key role in the formation of *P.aeruginosa* biofilms. Three main QS systems in *P.aeruginosa*: *las* system, *rhl* system and *Pseudomonas quinolone signal* system (PQS) encode for various signaling molecules that act as regulator for the transcription of numerous virulence factor genes (Zhang et al., 2013; Chadha et al., 2022). Zhao et al., conducted a study in which they screened DNA aptamers complimentary to the signal molecule C4-HSL of the *rhl* system. Depressing the *rhl* system affects the formation and maintenance of the biofilm. It was observed that the biofilm formation of *P.aeruginosa* was efficiently reduced to about 1/3 by the aptamers compared with that of the groups without the aptamers in the *in vitro* biofilm inhibition experiments (Zhao et al., 2019) (**Figure 4**).

Matchawong et al., constructed a 2’ -fluoropyrimidine modified nuclease-resistant RNA aptamersusing cell SELEX against *Streptococcus suis* serotype 2, strain P1/7. The R8-su12 RNA aptamer significantly reduced the *S. suis* biofilm formation and had the ability to bind to other pathogenic *S. suis* (serotype ½, 1, 9, and 14) (Matchawong et al., 2022). *Candida albicans* was grown in the exposure of condensed cigarette smoke (CSC), prepared from clove (CCSC) and non-clove (NCSC) cigarettes, for 48 h (Bachtiar et al., 2021).It was found that the presence of added CCSC or NCSC significantly enhanced *C. albicans* biofilm development but when *C. albicans* was precoated with aptamer (Ca-apt1) there was a significant impairment in the biofilm development accelerated by the NCSC and CCSC. This could be attributed to the enhancement of the morphological changes of *C. albicans* (from yeast to hypha formation) due to CCSC or NCSC was reduced due to precoating the aptamer.

Ning et al.,conducted an interesting study in which a GO-loaded aptamer/berberine bifunctional complex specific to penicillin-binding protein 2a (PBP2a) significantly inhibited MRSA biofilm formation (Ning et al., 2022). The aptamer blocks the function of PBP2a, reducing surface-cell attachment and berberine attenuates the level of the accessory gene regulator (agr) system, which is essential for MRSA biofilm formation. Furthermore, GO also has the potential to disrupt cell membranes, attributing to the antibiofilm activity (Saravanan et al., 2023). Lijuan et al**.,** developed an aptamer-ampicillin bifunctional conjugate that targeted bacterial flagella for treating biofilms (Lijuan et al., 2017).

## 6 Challenges and limitations

### 6.1 Stability and delivery concerns of aptamers

The term “steady state” describes the dynamic balance that results from consistent dosage between the total amount of a drug taken and its elimination. For aptamers, the steady-state or nearly steady-state concentration can be attained three to five times the half-life following aptamer administration, which has a steady-state duration of 17 to 29 hours (Lee et al., 2015). This emphasize on the importance of aptamer’s stability i.e.,The longer the aptamer stays in circulation for the treatment of infectious disorders, the greater the likelihood that it may encounter pathogens. The pharmacokinetic profile of the aptamer must be determined to proceed with trials, firstly the aptamer’s half-life *in vivo* is rather brief, lasting roughly 2 mins. Unmodified ssDNA oligonucleotides have a half-life of less than one minute (Griffin et al., 1993) (Sanjuán, 2012).

In conditioned media, HEK cells infected with *M. fermentans* exhibit ribonuclease activity that rapidly degrades RNA carrying 2′-fluoro- and 2′-O-methyl-modified pyrimidines. Similar ribonuclease activity was seen in a pure culture of *M.fermentans*, but not in a culture of uncontaminated HEK cells (Hernandez et al., 2012). RA-36, an aptamer with antithrombin properties, have shown rapid bloodstream elimination with a half life of 1 minute As opposed to 23 minutes in tissues (Zavyalova et al., 2017). The tissue type, aptamer sequence, and their formulation affects the outcomes of aptamer uptake ad distribution. A single bolus oral dose of aptamer was administered to mice, and after tissue contamination was eliminated with perfusion buffer, the aptamer was diffused into the bloodstream from the peritoneum and into multiple organs, including the brain and spinal cord, within minutes of oral administration. The uptake of the aptamer was reduced within a few hours (Perschbacher et al., 2015). When aptamer and nanoparticles are conjugated, the physiochemical properties including the size and distribution of the particles are altered (Ghassami et al., 2018).

### 6.2 Potential for off-target effects and safety issues

Drugs undergoing clinical trials may have side effects and safety problems of their own, but it is crucial to understand these effects in order to design a therapeutic version with less side effects. Investigations’ findings by Zhao et.al., in the tested settings, the SGC8 aptamer exhibited neither mutagenicity nor genetic toxicity using total body-positron emitting tomography (TB PET) (D. Ding et al., 2023). When an aptamer-tagged radioactive element was injected intravenously, the kidney contained the highest quantities of radioactivity. This indicates that the pharmacokinetics profile of absorption from intravenous aptamer injection results in a relatively low absorption rate. However, giving aptamer was administered in many doses, but this did not cause aptamer to accumulate in plasma. The factor most likely limiting the drug’s rate of disposal is its rate of absorption (Siddiqui and Keating, 2005).

Researchers discovered that the absence of conjugating cholesterol has certain undesirable effects, such as altering the expression of genes associated to innate immunity and cellular survival (Lee et al., 2015). In addition, the aptamer’s overall negative charge causes it to attach to positively charged substances without being specific. As stated previously, the aptamer’s short length and compact size promote bio clearance (Gholikhani et al., 2022).

### 6.3 Strategies to overcome challenges and ongoing research in the field

The aforementioned problems can be solved in a number of ways, and some of these tactics are (1) substituting sulfur for one of the monothio or dithio groups of the phosphoryl non-bridging oxygen atoms results in a number of benefits, including increased binding to the target, resistance to nuclease action, and faster absorption into the cells. However, there is a small drawback to this the aptamer may become less specific (Thiviyanathan et al., 2007; Keefe et al., 2010). (2) integrating aptamer into a larger molecular framework in the shape of a multivalent circle by offering nucleolytic stabilization that guards against exonucleases (Di Giusto et al., 2006). (3) the body’s nuclease enzymes could not break down aptamers synthesized with an L nucleotide sequence. This type of sequence, called spigelmers, is the mirror image of an oligonucleotide but contains L nucleotide instead of R nucleotide (Maasch et al., 2008; Chen et al., 2017). (4) RNA sequences that have aldehyde derivatives appended to the 5’ end, facilitate affinity purification and coupling with other molecules (Pfander et al., 2007). (5) producing the most stable hybrids by employing nucleoside analogues that have a methylene bond between the ribose ring’s 2′-O and 4′-C in order to create a locked nucleic acid sequence. The sugar moiety is thus locked in a C3′-endo configuration (Lebars et al., 2007). (6) an aptamer was modified by adding PEG linkers to decrease stearic hinderance and 2’-fluoro-pyrimidines (2’F) (Derbyshire et al., 2012; Ni et al., 2021). (7) the highest tissue exposure was achieved by the aptamer, which was prepared as a 3′ biotin derivative coupled with tetrameric streptavidin (Perschbacher et al., 2015). To achieve this, a cholesterol moiety was linked to the 5’ end of a 29-nucleotide RNA aptamer that had been modified with 2’-F against the HCV NS5B protein. This modification was chosen because previous studies have shown that conjugating oligonucleotide molecules with cholesterol can prolong their plasma half-life by associating with plasma lipoproteins and enhance their uptake by hepatic cells through receptor-mediated endocytosis. (8) An improved aptamer half-life results by conjugating cholesterol with the aptamer (Lee et al., 2015). (9) The aptamer attached to MetCyc, facilitating its interaction with other molecules helping evade attacks from nucleases (Borbas et al., 2007; Ni et al., 2021). (10) In some circumstances, we can employ liposomal conjugated aptamers to lengthen the drug’s half-life and make it more covert (Jiang et al., 2020; Alameh et al., 2021; Kim et al., 2001). (11) To shield the RNA from exonuclease degradation, the derivative’s two terminals are capped with an extended stem structure, allowing for the effective *in vivo* expression of the aptamer (Mori et al., 2012). Creating chimeric aptamers by combining segments from different aptamers or combining with other functional molecules for enhancing specificity (Cheng et al., 2023). In conclusion, these diverse strategies and modifications illustrate ongoing efforts to optimize aptamer technology, enhancing their stability, specificity, and therapeutic efficacy across various biomedical applications.

## 7 Conclusion and future perspectives

The diverse applications of aptamers in the realm of infectious diseases underscore their immense potential in diagnostics, therapeutics, and biosensing. The ability of aptamers to specifically recognize and bind to a wide range of pathogenic targets, including viruses, bacteria, and fungus, has paved the way for innovative solutions in disease detection and treatment. In diagnostics, aptamers have demonstrated exceptional sensitivity and specificity, enabling the development of rapid and accurate diagnostic assays. Their incorporation into biosensors has facilitated the detection of infectious agents at early stages, contributing to timely interventions and improved patient outcomes. Aptamer-based diagnostic platforms also offer the advantage of portability and cost-effectiveness, making them particularly valuable in resource-limited settings. Aptamers have proven their mettle in therapeutic applications, where they can be engineered to inhibit viral entry, replication, or modulate the host immune response. The versatility of aptamers allows for the design of tailored therapeutic interventions, offering a promising avenue for the development of antiviral and antibacterial agents. Moreover, the potential for aptamers to mitigate the emergence of drug-resistant strains adds another layer of significance to their therapeutic applications. Looking ahead, the future perspectives of aptamer research in infectious diseases are exciting and multifaceted. Advancements in aptamer selection technologies, such as SELEX, will likely enhance the discovery of aptamers with improved binding affinities and specificities. The integration of aptamers with emerging technologies, such as CRISPR-based diagnostics and gene editing, holds promise for the development of next-generation diagnostic and therapeutic tools. Furthermore, the exploration of aptamer-nanoparticle conjugates and other delivery systems may enhance the targeted delivery of aptamers to infected tissues, improving their therapeutic efficacy. Collaborations between academia, industry, and healthcare providers will be crucial in translating aptamer-based technologies from the laboratory to clinical practice.
